# Putative mapping of α-subunits in the human brain: A PET study of GABA _A_ receptor binding

**DOI:** 10.1162/imag_a_00464

**Published:** 2025-01-30

**Authors:** Zsolt Cselényi, Aurelija Jucaite, Lars Farde

**Affiliations:** PET Science Centre, Personalized Medicine and Biosamples, R&D, AstraZeneca, Stockholm, Sweden; PET Center, Department of Clinical Neuroscience, Center for Psychiatry Research, Karolinska Institutet, Stockholm, Sweden

**Keywords:** GABA _A_ receptor α-subunit, [ ^11^ C]flumazenil binding map, AZD7325, AZD6280, binding, occupancy

## Abstract

The benzodiazepines (BZ) bind to the GABA_A_receptor (GABA_A_R) at the interface of its α-/γ-subunits and exert pharmacological activity as allosteric modulators. However, the distribution of the six distinct α-subunits (α_1_–α_6_) in the human brain has not been mapped in detail, primarily due to lack of α-subunit selective radioligands. AZD7325 and AZD6280 were two drug candidates with partial α-subunit selectivity*in vitro*, in development for the treatment of anxiety. GABA_A_R occupancy of both drugs was examined in the human brain using [^11^C]flumazenil PET imaging, which visualizes GABA_A_Rs containing the α_1_-, α_2_-, α_3_-, or α_5_-subunits with similar sensitivity. Importantly, the pattern of occupancy was heterogeneous across brain regions and different between the two drugs. This observation encouraged us to extend the analysis in an attempt to generate tentative maps of α-subunits in the human brain.

Parametric images of [^11^C]flumazenil binding in 12 subjects, obtained at baseline and following administration of different doses of AZD7325 or AZD6280, were entered into a comprehensive analysis to identify GABA_A_R occupancy components of the two drugs. The major outcome parameters of the fitted models were maps of the contributions of these components to the overall occupancy and binding. The maps were then explored in terms of gross anatomy and were correlated with gene expression data for the relevant α-subunits to speculate on possible α-subunit identity of the derived components.

The overall occupancy was disentangled into three distinct components (C_1_to C_3_) by the preferred model. C_1_was occupied by both drugs, C_2_was only occupied by AZD7325, and C_3_was not occupied by either drug. The patterns of component-specific contributions were diverse and complex, dissimilar to each other and to the overall [^11^C]flumazenil binding. Of the three components, C_1_had the highest contribution throughout most of the brain except some cerebral nuclei, such as amygdala. The contribution of C_2_was notable in cortex and basal ganglia, and very low in thalamus and brain stem. Within the cortex, the contribution of C_3_was localized with highest values in sharply demarcated areas of the limbic, cingulate, and insular cortex. Otherwise, it had the highest contribution among components in some subcortical nuclei, was behind C_1_in thalamus, and was negligible in brain stem. All three components had a high-degree, statistically significant positive correlation with GABA_A_R α-subunit gene (GABRA) expression: C_1_foremost with GABRA1, C_2_foremost with GABRA2, and C_3_foremost with GABRA5. The correlations suggest that C_1_might correspond to the distribution of α_1_- (and possibly α_3_-), C_2_to that of α_2_-, and C_3_to that of α_5_-subunit-containing GABA_A_Rs, respectively.

The components identified by the present analysis of occupancy patterns at the [^11^C]flumazenil binding site provided putative*in vivo*maps of α-subunit-specific GABA_A_R distribution in the human brain. The findings demonstrate the feasibility of developing small molecules having preference for certain α-subunits, even if full selectivity was not yet achieved. Accordingly, the results should encourage and support the development of optimized, fully selective compounds to the benefit of basic research and drug development for the treatment of neurological and psychiatric conditions.

## 1 Introduction

The benzodiazepines (BZs) were introduced in the early 1960s and became widely used for their anxiolytic, sedative, anticonvulsant, and muscle relaxant effects. With time it became obvious that the BZs carried concomitant undesirable effects such as dependence and overdose risk. For that reason, numerous attempts have been made to develop safer BZs.

The BZs were originally developed purely from empirical observations (Wick, 2013), whereas the pharmacological target at the γ-aminobutyric acid (GABA) system was not identified until the late 70s (Braestrup & Squires, 1977;Haefely et al., 1975). Now we know that the GABA_A_receptor (GABA_A_R) is a pentameric ligand-gated ion channel assembled from the known 19 pore-forming subunits (subunit families α, β, γ, δ, ε, θ, and ρ) with a most typical stoichiometry of two α-, two β-, and one γ-subunit (Kim & Hibbs, 2021;Olsen & Sieghart, 2008;Sieghart, 1995). Importantly, BZs serve as allosteric modulators at the α-subunit. This new understanding raised the hypothesis whether GABA_A_R-mediated therapy can be optimized by the development of α-subunit selective drugs (Rudolph et al., 1999). However, despite considerable industrial efforts, the development of such drugs has had limited success. In addition, there is lack of radioligands that are selective for distinct α-subunits, thus limiting the tools for direct*in vitro*/*in vivo*confirmation of subunit selectivity.

Flumazenil is a nonselective GABA_A_R antagonist at the BZ site, with equipotent affinity for the benzodiazepine-sensitive GABA_A_Rs (i.e., those containing the α_1_, α_2_, α_3_, or the α_5_GABA_A_R subunits) and two orders of magnitude lower affinity for those with the α_4_- and α_6_-subunits (Sieghart, 1995). Flumazenil was early on labeled with carbon-11 for molecular imaging by positron emission tomography (PET) (Persson et al., 1985) and has been broadly applied for imaging of the GABA_A_R and recently for detailed mapping of GABA_A_R binding in the human brain (Nørgaard et al., 2021). An initial step toward selective visualization of α-subunits was taken by the development of [^11^C]RO15-4513, a radioligand partially selective for the α_5_- subunit (Halldin et al., 1992;Myers et al., 2017). However, due to the mentioned lack of selective radioligands, the distribution of the other α-subunits in the human brain*in vivo*remains to be fully understood.

**Fig. 1.**
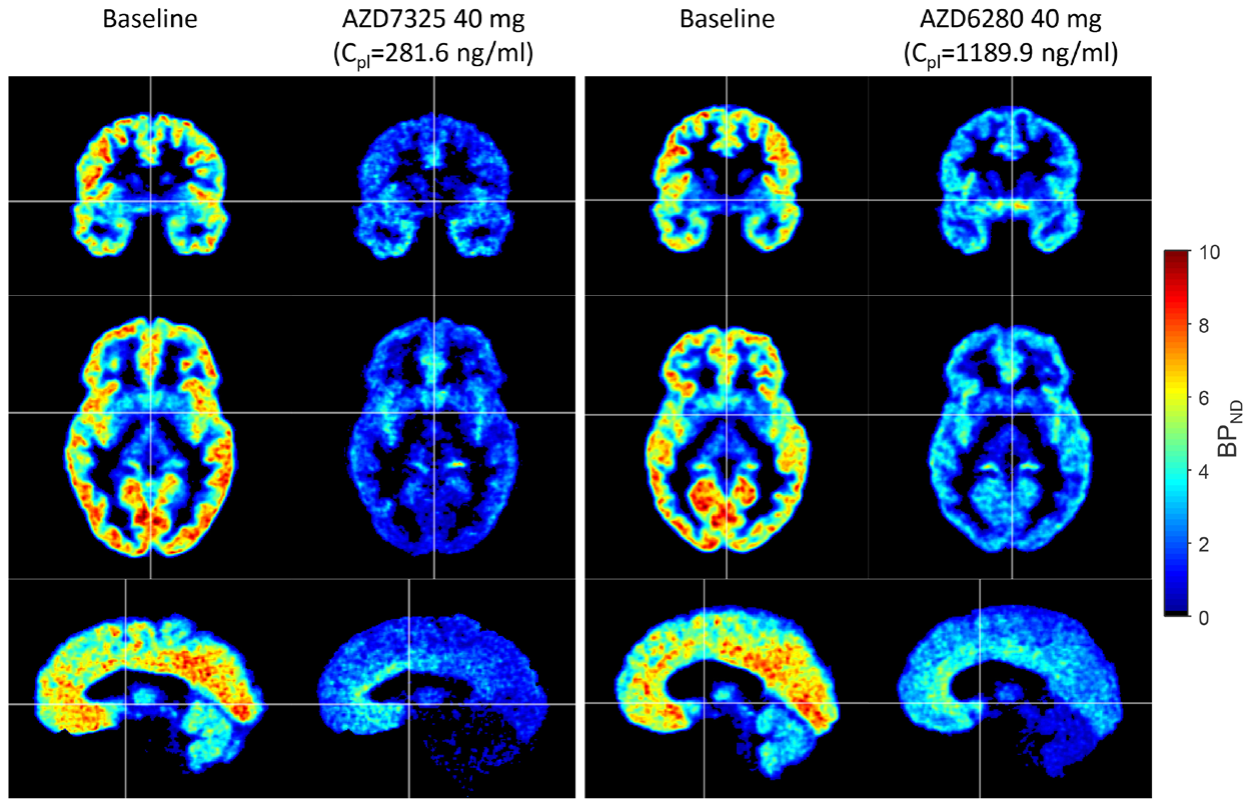
Parametric images showing orthogonal brain sections of [^11^C]flumazenil binding (BP_ND_) at baseline and following drug administration in two subjects (Jucaite et al., 2017). The panel on the left is for the subject who had the highest plasma exposure of AZD7325 and the panel on the right is for the subject who had highest plasma exposure of AZD6280. Crosshairs indicate the location of the orthogonal sections.

A decade ago, AstraZeneca Pharmaceuticals developed two GABA_A_R partial agonists at the BZ site intended for the treatment of anxiety (Chen et al., 2014,^2015^). AZD7325 has high*in vitro*affinity for the α_1_-, α_2_-, and α_3_-subunits (K_i_values from 0.3 to 1.3 nM) while AZD6280 is more selective and has high affinity to the α_1_-subunit (K_i_of 0.5 nM) and lower affinity to the α_2_- and α_3_-subunits (K_i_of 21 and 31 nM, respectively). Both drug candidates have low affinity for the α_5_-subunit containing GABA_A_receptors (K_i_of 230 and 1680 nM, respectively). The development program included two PET studies on drug-induced occupancy at the [^11^C]flumazenil binding sites in the human brain (Jucaite et al., 2017). Importantly, the pattern of occupancy was heterogeneous across brain regions and different between the two drugs (Fig. 1).

The demonstrated differences between the regional occupancy patterns encouraged us to extend the PET image analysis regarding the level of anatomical and explanatory detail. The first key condition for this analysis is that occupancy at a given α-subunit-containing GABA_A_R subtype is expected to be the same across brain regions, though different for each drug and dose. This condition is supported by the literature indicating that the majority of benzodiazepine-sensitive GABA_A_R subtypes in brain comprise α_1/2/3/5_β_1–3_γ_2_pentamers (Olsen & Sieghart, 2008;Sieghart & Sperk, 2002). Importantly, [^11^C]flumazenil has comparable affinities for all these subtypes, which in turn suggests that its PET signal is likely dominated by these GABA_A_Rs (Sieghart, 1995). In other words, [^11^C]flumazenil has lower affinities for other GABA_A_R subtypes and such binding is thus not likely contributing to the PET signal. Furthermore, it was earlier reported that for benzodiazepines in general, there are no major affinity differences as a function of the constituent β subunit, only as a function of the α-subunit (Sieghart, 1995).

The second condition is that the expression of each α-subunit varies across the brain with detectable contribution to the PET signal in several regions and is not dependent on drug or drug dose and likely similar across subjects. An indirect support for this condition is the previously published data on GABA_A_R subunit gene expression that showed that proportional gene expression of each of the 4 α-subunits found in BZ receptors reaches at least ~10% in at least 25% of brain regions relative to the summed expression of these 4 subunits (Sequeira et al., 2019). Taking advantage of these two conditions, the dataset, obtained on a high-resolution PET system, should allow for disentangling the observed [^11^C]flumazenil binding and occupancy into constituents/components, which are differently bound and occupied by AZD7325 and AZD6280, with considerable anatomical precision across the brain. This analytical approach is reminiscent of prior published works using heterologous competition data: for example, where the binding of the purported mGluR5 radioligand [^11^C]AZD9272 has been decomposed into MAO-specific and mGluR5-specific components (Varnäs et al., 2020), or where the α_5_-subunit-containing GABA_A_R selectivity of [^11^C]Ro15-4513 was quantified (Myers et al., 2017).

The aim of the present image analysis was to capture and quantify the differences in drug-specific regional [^11^C]flumazenil occupancy patterns in more detail. In total, 12 subjects had been examined and parametric images of [^11^C]flumazenil binding had been obtained at baseline and after administration of different doses of AZD7325 or AZD6280 (Jucaite et al., 2017,Fig. 1). Parametric images were entered into a comprehensive analysis to obtain maps of the fractional contribution of components of [^11^C]flumazenil binding across the brain. In addition, recently published GABA_A_R subunit gene expression data (Hawrylycz et al., 2012;Sequeira et al., 2019) were used to explore the components and interpret them in terms of α-subunit identity.

## 2 Methods

### 2.1 Participants

The present image analysis was based on two previous PET studies sponsored by AstraZeneca (ClinicalTrials.gov: NCT00681720 and NCT00681746). The experimental procedure and results have been published in detail (Jucaite et al., 2017). Written informed consent was obtained from participants before the initiation of the studies. Both studies were approved by the Regional Ethical Review Board in Stockholm, and the Radiation Safety Committee at Karolinska University Hospital (KUH), Stockholm, Sweden.

### 2.2 Image acquisition

Study participants were recruited at the AstraZeneca CPU, Karolinska University Hospital (KUH), Huddinge. Prior to PET measurements, 3D brain MRI examinations were acquired on a 1.5-T General Electric Signa system (GE, Milwaukee, WI, USA) at the MRI Center of KUH, Solna. Two examinations were made in one session. The T2-weighted images were acquired for clinical evaluation, and the T1-weighted images were used as anatomical reference for analyzing the PET images. The T1-weighted sequence was a 3D SPGR protocol in the axial plane with the following parameters: TR 23 ms, TE 4 ms, matrix 256 × 192 × 156, and voxel size 1.02 × 1.02 × 1.0 mm. PET imaging was performed at the PET Centre, Department of Clinical Neuroscience, Karolinska Institutet, Stockholm, Sweden. The binding of the radioligand [^11^C]flumazenil was estimated at baseline conditions and after drug administration. Single oral doses of AZD7325 (0.2, 1, 2, 5, 20, 30 mg) and AZD6280 (5, 12, 20, 30, 40 mg) were administered and PET data were obtained over 63 minutes with a HRRT PET system (Siemens/CTI, Knoxville, TN, USA) (for full details on subjects and doses, seeJucaite et al., 2017). The plasma concentration of tested drugs during PET acquisition was determined by solid-phase extraction using Waters HLB Elution plate followed by LCMS/MS (AstraZeneca, DE, USA).

### 2.3 Initial PET image analysis

In the previously reported analysis, parametric images showing the binding potential (BP_ND_) were generated using wavelet-aided parametric imaging (WAPI) (Cselényi et al., 2006). This approach included the multi-linear, reference input version of Logan’s graphical analysis using the time–radioactivity curve (TAC) of pons as input. Pons was used as reference region of interest (ROI). The parametric images were used as “input data” in the present analysis (see sample images inFig. 1). The analysis was implemented in MATLAB® R2022b (The Mathworks, Natick, MA, USA) with some steps carried out in FreeSurfer (version 7.2.0) as indicated below (Fischl, 2012).

### 2.4 Extended image analysis: Major parts

**Fig. 2.**
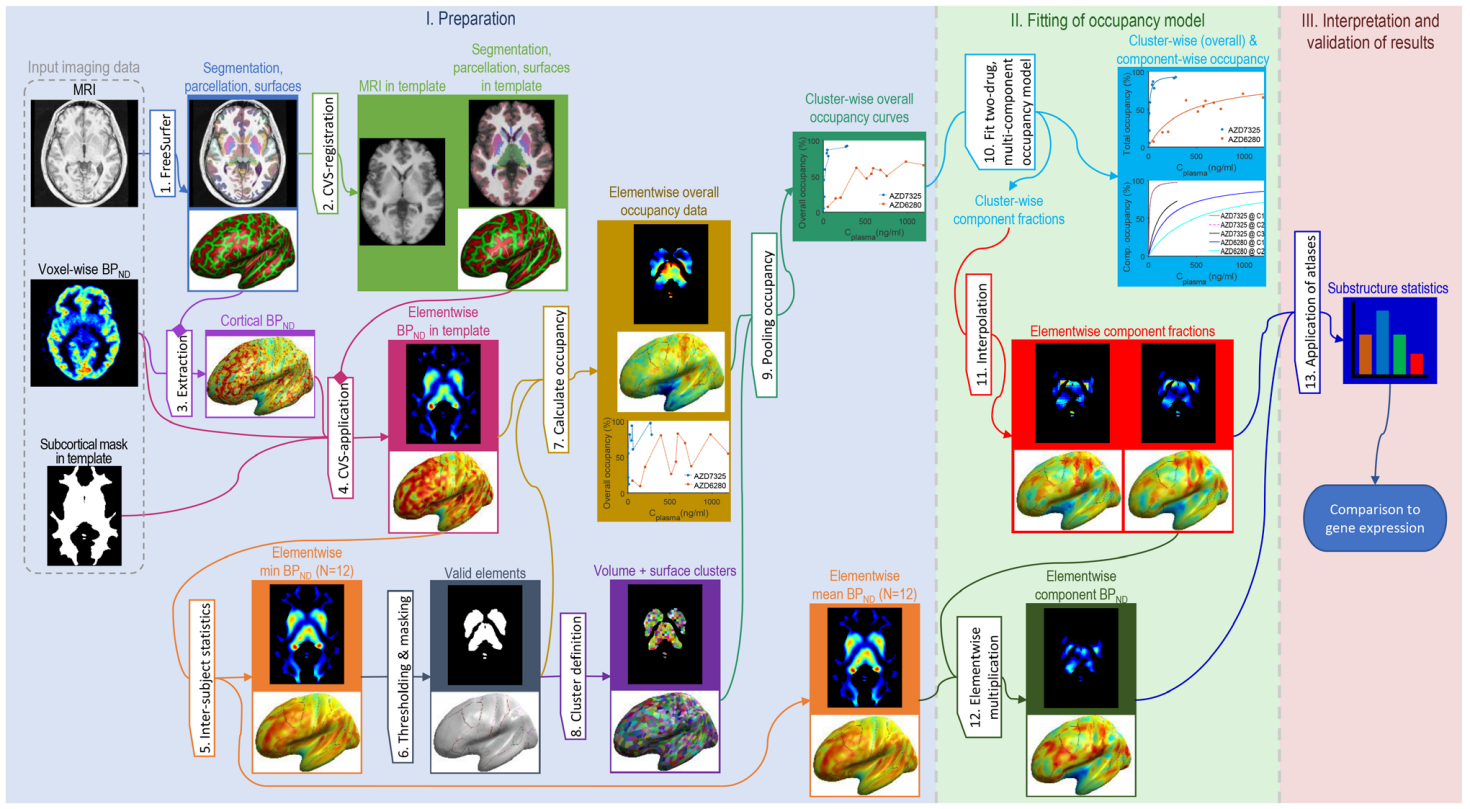
Flow chart of the three major parts (I–III) of the image analysis process.

MR images and BP_ND_parametric images were entered into an extended analysis, which consisted of three major parts (Fig. 2):

**I. Preparation.**To enable a comprehensive analysis, individual imaging data were projected into a common neuroanatomical template space accounting for interindividual differences in cortical folding patterns and subcortical structures. The main output of this part was overall [^11^C]flumazenil occupancy in template space.

**II. Fitting of occupancy model.**The aim of this part was to generate maps showing the distribution of drug-displaceable and non-drug-displaceable components of [^11^C]flumazenil binding. A series of tailor-made occupancy models, having up to four binding components (C_1_–C_4_), were fitted to parametric maps of overall occupancy for both drugs at the same time. In particular, the brain region-specific overall measured occupancy was fitted as the weighted sum of the component-specific occupancies where the weighting factors were the fractional contributions of the model-derived components. As pointed out in the introduction, the component- and drug-specific occupancies are not dependent on location in brain, whereas the regional expression levels are location specific. The fitting procedure in part II yielded maps of the components’ fractional and absolute contributions. A preferred model variant was selected based on model performance.

**III. Interpretation and validation of results.**To summarize and explore the derived maps, gross anatomical brain atlases were applied. Furthermore, to explore the biological validity and underpinnings of the component maps, the patterns of contributions were compared with gene expression data of the relevant GABA_A_R α-subunits.

### 2.5 Extended image analysis: Detailed description

The detailed steps of the extended analysis are illustrated inFigure 2and described below where the label and numbering of each step match between description and figure:

- **Preparation**
  1. FreeSurfer:
    The individual reoriented MR images were processed in the FreeSurfer (version 7.2.0) analysis pipeline to obtain cortical surface extraction and anatomical parcellation as well as subcortical segmentation (Fischl, 2012).
  2. Combined Volumetric and Surface (CVS) registration:
    The results of cortical parcellation and subcortical segmentation were for each subject entered for atlas registration using the CVS registration algorithm in FreeSurfer (Postelnicu et al., 2009). The method accounts for interindividual overall and local volumetric differences in brain shape, nuclei, and other subcortical brain structures as well as cortical folding patterns. The CVS template in the Montreal Neurological Institute (MNI) atlas space (cvs_avg35_inMNI152) was used as target of the registration. Thereby, the CVS registration provided the parameters necessary for warping arbitrary volumetric and surface data from the individual anatomical to the MNI152 atlas space. Note that the FreeSurfer processing in step 1 excluded the hippocampus from the cortical surface. However, the cortical surface in the cvs_avg35_inMNI152 CVS template (originally derived in FreeSurfer version 6.0) cuts into the hippocampus and amygdala. Since this discrepancy would lead to erroneous hippocampal CVS registration, we performed cortical surface extraction, parcellation, and segmentation of the cvs_avg35_inMNI152 template image in FreeSurfer vesion 7.2.0 before step 2 (this version of the template can be found online athttps://doi.org/10.12751/g-node.83wfme).
  3. Extraction:
    FreeSurfer provided the inner (toward white matter) and outer (toward pia mater) surfaces of the cortical gray matter, which were used to extract flumazenil binding potential (BP_ND_) data for cortex. In detail, each surface vertex in the “white” surface mesh has a corresponding vertex in the pial surface with a line connecting the two that is exactly perpendicular to the tangential plane of the pial cortical surface at that point. Accordingly, for each pair of vertices, the parametric image was sampled along the connecting line at a fractional distance of 0.4 to 0.6 (in 0.02 increments) between the points with the mean value of these 11 samples taken as the corresponding surface BP_ND_. The conservative sampling around the middle point across the thickness of the cortical ribbon was applied to minimize the impact of partial volume effects in the PET parametric images.
  4. CVS application:
    Using the CVS registration parameters, the individual volumetric and cortical surface BP_ND_data were warped to the cvs_avg35_inMNI152 template space. A binary image mask of subcortical voxels in MNI152 space was used to retain subcortical voxels in the volumetric portion of the binding data. As noted above (step 2), hippocampus was assigned to the volumetric, “subcortical” data in FreeSurfer and not to the cortical ribbon. Both volumetric and surface data were then smoothed with a Gaussian filter (2D or 3D, respectively) using a kernel with a full width at half maximum (FWHM) of 5 mm. Several of the steps below operated on such data at a volumetric level for subcortical voxels or at a surface level for cortical vertices. The expression “elementwise” refers to this collection of volume and surface data points.
  5. Inter-subject statistics:
    For each baseline BP_ND_element, the lowest value (minimum) and the mean (N = 12) were calculated.
  6. Thresholding & masking:
    A binary mask of “valid” data elements was obtained by thresholding the inter-individual minimum elementwise BP_ND_data at 0.1 (inclusive). Furthermore, for the volumetric part excluding the cortex, only voxels within the following anatomical regions were retained: amygdala, hippocampus, striatum, pallidum, thalamus, ventral diencephalon, brain stem, and cerebellar cortex (the non-cortical volumetric mask can be found online athttps://doi.org/10.12751/g-node.83wfme).
  7. Calculation of occupancy:
    Elementwise overall occupancy, that is, reduction in [^11^C]flumazenil binding, was calculated for each pretreatment PET measurement according to the following equation:
$$\begin{array}{l} Occ_{i,j}^{tot}=1-\frac{BP_{ND_{PT}\_i,j}}{BP_{ND_{BL}\_i,j}}|1\leq i\leq M_{VS};\\ j\in \left\{AZD7325,AZD6280\right\}, \end{array}$$ (1)
    where*i*is indexing elements with M_VS_equal to the number of valid volume and surface elements (507690),*j*refers to the drug (AZD7325 or AZD6280),$BP_{ND_{BL}\_i,j}$and$BP_{ND_{PT}\_i,j}$are the baseline and pretreatment binding potentials for drug*j*at element*i*. Note that indexing by occasion (drug dose) is omitted for clarity.
  8. Cluster definition:
    To reduce the computational burden of the subsequent non-linear modeling step, and to reduce the variance of the elementwise overall occupancy data, due to inter-individual variability, test–retest variation and statistical noise, the elements were assigned to geometrically defined clusters or neighborhoods. In detail, voxels in 4 × 4 × 4 blocks were assigned to the same cluster. This step corresponds to a 64-fold data reduction, that is, with a Euclidean distance less than 4 mm from the cluster centroid for each voxel. Analogously, neighboring vertices were assigned to clusters leading to a ~120-fold surface data reduction. Here, the surface clusters were obtained by a geometry-preserving reduction of the vertices of the inflated, spherical representation of the cortical data such that ~120 vertices within small hexagonal patches across the surface were assigned to the same cluster with an average Euclidean distance of ~4 mm of the vertices to the cluster centroid. Following this reduction, the total number of clusters was 6469, that is, far less than the original 507690 elements.
  9. Pooling of occupancy values:
    The mean elementwise overall occupancy data were calculated across cluster members for each cluster and drug pretreatment occasion (i.e., yielding cluster-wise$Occ_{i,j}^{tot}$with*i*now indexing clusters).
- **Fitting of occupancy model**
  10. Fit of two-drug, multi-component occupancy models:
    Initially, several multi-component model configurations were fitted and compared as described inSection 2.5.1below. See alsoSupplementary Figure S1for a schematic view of the tested model configurations. Based on model comparisons, a preferred model configuration was finally selected.
    Each model was fitted to the cluster-wise$Occ_{i,j}^{tot}$and drug plasma exposure (C_p_) data and was based on the following set of equations:
$$\begin{array}{l} Occ_{j,k}=\frac{C_{p\_j}}{C_{p\_j}+K_{i\_j,k}}|j\in \left\{AZD7325,AZD6280\right\};\\ 1\leq k\leq N_{j};N_{j}\leq N-1, \end{array}$$ (2)
$$\begin{array}{l} \hat{Oc}c_{i,j}^{tot}=\sum_{k=1}^{N_{j}}f_{i,k}\times Occ_{j,k}|1\leq i\leq M_{Cl};\sum_{k=1}^{N}f_{i,k}=1, \end{array}$$ (3)
    where*i*is indexing clusters with M_Cl_equal to the number of clusters (6469),*j*refers to the drug (AZD7325 or AZD6280), N_j_is the number of displaceable components of [^11^C]flumazenil’s binding for drug j, N is the total number of components of [^11^C]flumazenil binding including a non-drug-displaceable component,$Occ_{j,k}$is the occupancy of drug*j*at displaceable component*k*of flumazenil binding,$C_{p\_j}$is the plasma exposure of drug*j*at each pretreatment occasion (indexing by occasion is omitted for clarity),$K_{i\_j,k}$is the dissociation constant of drug*j*at binding component*k*,$\hat{Oc}c_{i,j}^{tot}$is the model-predicted overall occupancy (reduction in radioligand binding) for cluster*i*and drug*j*, and$f_{i,k}$is the fractional contribution of component*k*to [^11^C]flumazenil binding in cluster*i*. As already pointed out, the component-specific occupancies are not location dependent, while the component-specific fractional contributions to [^11^C]flumazenil binding are not drug dependent. Using this model, the best-fit values of$K_{i\_j,k}$and$f_{i,k}$were estimated using non-linear least squares optimization, that is, minimizing the sum of squared residuals:$\sum_{i=1}^{M_{Cl}}\sum_{j}{\left(Occ_{i,j}^{tot}-\hat{Oc}c_{i,j}^{tot}\right)}^{2}$.
    Instead of directly fitting the component-specific fractional contributions ($f_{i,k}$) according toEquation 3, a change of variables$f_{i,k}\to v_{i,k}$was applied using the following equations to avoid generation of nonsensical values:
$$v_{i,k}=\frac{\sum_{q=1}^{k}f_{i,q}}{\sum_{q=1}^{k+1}f_{i,q}}|1\leq k<N;0\leq v_{i,k}\leq 1,$$ (4)
    and its inverse:
$$f_{i,k}=\prod_{q=k}^{N}v_{i,q}-\prod_{q=k-1}^{N}v_{i,q}|1\leq k\leq N;\;v_{0}\equiv 0;\;v_{N}\equiv 1.$$ (5)
    Accordingly, with the fitted$v_{i,k}$parameters (1≤k<N) restricted to fall between 0 and 1 (inclusive), the corresponding$f_{i,k}$parameters were ensured to be non-negative and sum to unity (1≤k≤N)as required by the modelEquation 3.
    The starting values for the component-specific affinity values (or rather$K_{i\_j,k}$parameters) were such that the higher affinity values (lower$K_{i\_j,k}$) were given to the lower order components. The$v_{i,k}$parameters were initialized by obtaining them according toEquation 4from initial$f_{i,k}$set to 1/N.
  11. Interpolation:
    The cluster-wise fractional contributions from the components were projected to the level of elements in two ways. In the first way, supporting the comparison with regional gene expression (step 13b), the elementwise fractional contributions were obtained by simply assigning the corresponding cluster’s result. In the second way, appropriate for visualization and derivation of absolute contribution maps (step 12), the cluster-wise component fractional contributions were assigned to the level of elements using linear interpolation. Trilinear interpolation was used in the volumetric (subcortical) part, and bilinear interpolation was used in the surface (cortical) part obtained from FreeSurfer’s spherical representation of the cortical data.
  12. Elementwise multiplication:
    The inter-subject mean elementwise BP_ND_data set (obtained in step 5) was multiplied with the linearly interpolated elementwise component-specific fractional contribution parameters to obtain the elementwise component-specific absolute contributions expressed as BP_ND_s.
- **Interpretation and validation of results**
  13. Application of atlases and comparison with gene expression. The region (structure) definitions of two anatomical atlases were used for visualization and to calculate regional average values in the contribution maps. In detail:
    - For gross anatomical designation of the observed component-specific cortical and subcortical patterns, the Desikan–Killiany atlas’s surface parcellation and the FreeSurfer’s subcortical segmentation were used, respectively.
    - To obtain a more detailed anatomical designation and for comparison with GABA_A_R gene expression data, the 3D Allen Human Reference Atlas (AHRA) was applied (Ding et al., 2020,http://download.alleninstitute.org/informatics-archive/allen_human_reference_atlas_3d_2020/version_1/). The brain regions of this atlas are available as a volumetric label of the MNI152NLin2009bSym template (ICBM 2009b Nonlinear Symmetric) brain image (Fonov et al., 2009) and comprise 141 substructures including gray and white matter substructures (129 left/right mirrored and 12 midline structures). While the subcortical structures are in close register between the MNI152NLin2009bSym and cvs_avg32_inMNI152 templates, the cortical folding patterns are slightly different. Accordingly, the AHRA volumetric labels could be used directly for subcortical structures, but the labeling of cortical structures in the cvs_avg32_inMNI152 template required additional steps. First, the MNI152NLin2009bSym T1-weighted MR image was processed in FreeSurfer (version 7.2.0) to obtain the cortical surface for this template. The cortical surface labels for the Allen Human Reference Atlas (AHRA) were then extracted by sampling the volumetric labels along the cortical ribbon of the MNI152NLin2009bSym template. Following spherical registration of the cortical surfaces of the two templates in FreeSurfer, the labels were finally transferred to the cortical surface of the cvs_avg32_inMNI152 template. The complete surface and volumetric label set contained 270 distinct regions of interest (ROIs) (2 × 129 side-specific and 12 midline ROIs), of which 221 labeled ROIs represented gray matter (2 × 108 side-specific and 5 midline ROIs, i.e., 113 ROIs with left-right pooling). The surface and volumetric label files and the list of ROI names can be found online athttps://doi.org/10.12751/g-node.83wfme.

Regional microarray expression data were obtained from an analysis of 6 post-mortem brains (1 female, 5 male, ages 24.0–57.0, 42.50 ± 13.38 years) provided by the Allen Human Brain Atlas (AHBA,https://human.brain-map.org) (Hawrylycz et al., 2012). Microarray data were processed using an augmented variant of the*abagen*software toolbox (based on version 0.1.4 + 15.gdc4a007;https://github.com/rmarkello/abagen) (Arnatkevic̆iūtė et al., 2019;Markello et al., 2021). To extract regional gene expression data, a curated volumetric version of the AHRA atlas in MNI152NLin2009bSym space was obtained, with labels for 98 distinct substructures (covering all 221 gray matter ROIs considering left–right pooling and pooling 2 × 23 ROIs belonging to 8 substructures that allowed for better sample coverage in the gene expression data, seeSupplementary File 2). We employed the microarray probe selection for GABA_A_R genes using a previously published approach (Sequeira et al., 2019). The tissue samples metadata in the microarray data include the anatomical substructural designations provided by the AHBA ontology (see the coronal AHBA sections athttps://atlas.brain-map.org/atlas?atlas=265297125), as well as MNI 3D coordinates. The MNI coordinates of tissue samples were updated to those generated via non-linear registration using the Advanced Normalization Tools (ANTs;https://github.com/chrisfilo/alleninf). To increase spatial coverage, tissue samples were mirrored bilaterally across the left and right hemispheres (Romero-Garcia et al., 2018). The original abagen software performs sample assignments to brain regions based on sample MNI coordinates by identifying the atlas parcel containing (or nearest to) the given sample. To reduce the potential for misassignment, the matching in the original version is constrained at the gross anatomical structural level (such as cortex and subcortex).

However, thanks to the 3D AHRA, which was made available following the generation of the original abagen software, we were able to implement a more fine-grained approach for constraining sample-to-region matching, further minimizing the possibility of sample misassignment. Importantly, there is a close correspondence of the AHRA and AHBA ontologies at the substructural level, in terms of both structure terminology and boundaries between corresponding substructures (confirmed by inspecting the 3D AHRA volume and the coronal sections for AHBA). Accordingly, for each of the 98 curated 3D AHRA substructures matching AHBA substructures could be identified. In most cases, an equivalent AHBA substructure or set of AHBA substructures were found. In a few cases, two or three AHRA substructures together were covering an equivalent AHBA substructure. The master table showing the mapping between curated AHRA substructures (with their constituent AHRA ROIs) and the corresponding AHBA substructures is found inSupplementary File 2. Based on this mapping, the augmented abagen sample assignment process restricted the sample MNI coordinate mapping to those atlas parcels which had a corresponding AHBA substructure containing the given sample according to their original anatomical designation. In other words, samples that had a one-to-one mapping between the AHRA and AHBA nomenclatures were directly assigned to the corresponding AHRA substructure. Samples with multiple AHRA correspondences, however, were assigned by abagen to the correct AHRA substructure that was closest based on their MNI coordinates. All tissue samples not assigned to an AHRA substructure were discarded. Samples assigned to the same AHRA substructure were averaged separately for each donor and then across donors yielding a single average value for each gene per substructure. Only regions containing gene expression data for all 6 donors and valid regional component contribution parameters were included in the comparison (62 substructures).

The regional patterns of component contributions were compared visually with the processed GABA_A_R α-subunit gene expression data and by calculating Pearson product-moment correlation coefficients (R values). The degree (size) of positive association was categorized according to the following cutoffs for the R value: R ≤ 0.0—lack of positive correlation; 0.0 < R ≤ 0.3—negligible positive correlation; 0.3 < R ≤ 0.5—low positive correlation; 0.5 < R ≤ 0.7—moderate positive correlation; 0.7 < R ≤ 0.9—high positive correlation; 0.9 < R ≤ 1.0—very high positive correlation. Note that only positive correlations are of interest in exploring the α-subunit identity of the components. The p-values for the correlations were corrected for multiple comparisons using the Benjamini–Hochberg false discovery rate (FDR) procedure (Benjamini & Hochberg, 1995). In detail, the obtained regional gene expression data for GABRA1, GABRA2, GABRA3, and GABRA5 genes, that is, the genes corresponding to the four GABA_A_R subtypes recognized by flumazenil, were first converted to fractional units by dividing the gene-specific expression with the summed expression of the four genes (non-log2-transformed). In this way, the gene expression of interest was quantified in a manner commensurate with the primary outcome parameters of the present PET analysis, the component-wise estimated regional fractional contributions. Note that while the selection of the preferred model was prior to, that is, did not rely on the comparison with gene expression data, yet the observed correlations for the selected model were subjected to sensitivity analysis by calculating the correlations for the cross-validation data sets first used in the model selection (described inSection 2.5.1).

#### 2.5.1 Strategy for model configurations and selection of a preferred model

As outlined in step 10 above, multiple occupancy model configurations were fitted to the data (see schematic view of model configurations in the top panel ofSupplementary Fig. S1with detailed description in the figure legend). The initial choice of model configurations was guided by the binding characteristics*in vitro*of the radioligand [^11^C]flumazenil as well as the two drug candidates AZD7325 and AZD6280 (values shown in the introduction). In detail, the main guiding principles were that

- [^11^C]flumazenil is expected to bind more subtypes than either drug, resulting in an overall displaceable and non-displaceable component of its binding.
- In the drug-displaceable portion, AZD7325 may be able to occupy more components than AZD6280.
- AZD6280 should have a maximum of one high affinity component and potentially one lower affinity one.
- Maximum three drug-displaceable components are possible with AZD7325 having high affinity for at least two and AZD6280 having high affinity for one.

Accordingly, a total of two to four model components were considered including one that was not displaced by either drug. The two-component model (M2a) assumed that only the lumped occupancy can be quantified, representing the high-affinity portions of drug binding. In all model configurations with three or four components, both drugs were assumed to have at least one high-affinity component with detectable occupancy. The four-component models assumed that specific binding at all three drug-recognizable α-subunits (α_1_, α_2_, α_3_), and thus their respective occupancy components could be disentangled.

The models were compared with the aim of identifying a preferred configuration. However, while in typical PET quantification the preference is established through purely statistical means, the current work relied on multiple means of evaluating model performance. The present evaluation still included the statistical assessment of the goodness of the fit. However, it also included parameter sanity and model generalizability checks.

The goodness of fit statistical parameters included the final sum of squared residuals, mean squared error, Akaike’s Information Criterion, and pair-wise F-tests between all possible pairs of less and more complex model configurations.

The parameter sanity and model generalizability checks included (1) calculating the percent of zero fitted fractional contribution parameters, (2) cross-validation (sensitivity analysis), and (3) checking parameter asymmetry. The fraction of zero fitted fraction contribution parameters was calculated since a model with relatively more zeros may indicate an overfit to the data.

For cross-validation (CV), each model was repeatedly fitted to a subset of the data, in each CV subset excluding one subject’s data from the AZD7325 and one from the AZD6280 experiments, respectively. All possible subject pairings were considered for exclusion in turn, that is, in total 32 CV models were fitted. Then, for each CV model-, cluster-, and component-specific fractional contribution value, the difference from the full model’s respective value was calculated. The median absolute difference was used as an index of the stability (variability) of the given model configuration.

Asymmetry was assessed in two steps. First, asymmetry was calculated at the level of brain regions (according to the Desikan–Killiany cortical parcels and FreeSurfer subcortical segments) by taking the absolute difference between the left and right side regional fractional contribution values in percentage of the mean of the two values. Second, the overall asymmetry for the brain was obtained by taking the median asymmetry value across regions. Note that prior studies have shown that the overall [^11^C]flumazenil binding is symmetric (Nørgaard et al., 2021), which was confirmed in the present data set (median absolute asymmetry was 4%). Thus, it appeared reasonable that subtype-specific components of [^11^C]flumazenil binding should also be symmetric.

## 3 Results

### 3.1 Multi-component occupancy model fitting and model selection

Initially, the PET parametric and MR images were processed to obtain elementwise occupancy maps in the cvs_avg35_inMNI152 template space. Several multi-component model configurations were then fitted to the prepared occupancy data (seeSupplementary Fig. S1, supplementary figures and tables are found inSupplementary File 3). The model variant M3c was the overall best considering statistical performance, parameter sanity, and model generalizability checks. In detail, M3c was among the top 3 variants across all metrics of model performance. According to F-tests, model M3c was superior to the three simpler model variants (M2a, M3a, and M3e) and to most of the more complex model variants (Supplementary Table S1). The exceptions were model M3d and M4d of which M3d was superior in all F-tests. However, while M3d was preferred according to purely statistical metrics, it was behind M3c in sanity and model generalizability checks as it had a higher proportion of zero parameters, higher cross-validation error, and on average higher parameter asymmetry. Similar considerations applied to model M4d as well. Thus, in contrast to model M3c, models M3d and M4d appeared to end up overfitting the data with lower generalizability despite their more favorable statistical performance. Taken together, the M3c variant was selected as the preferred model.

**Fig. 3.**
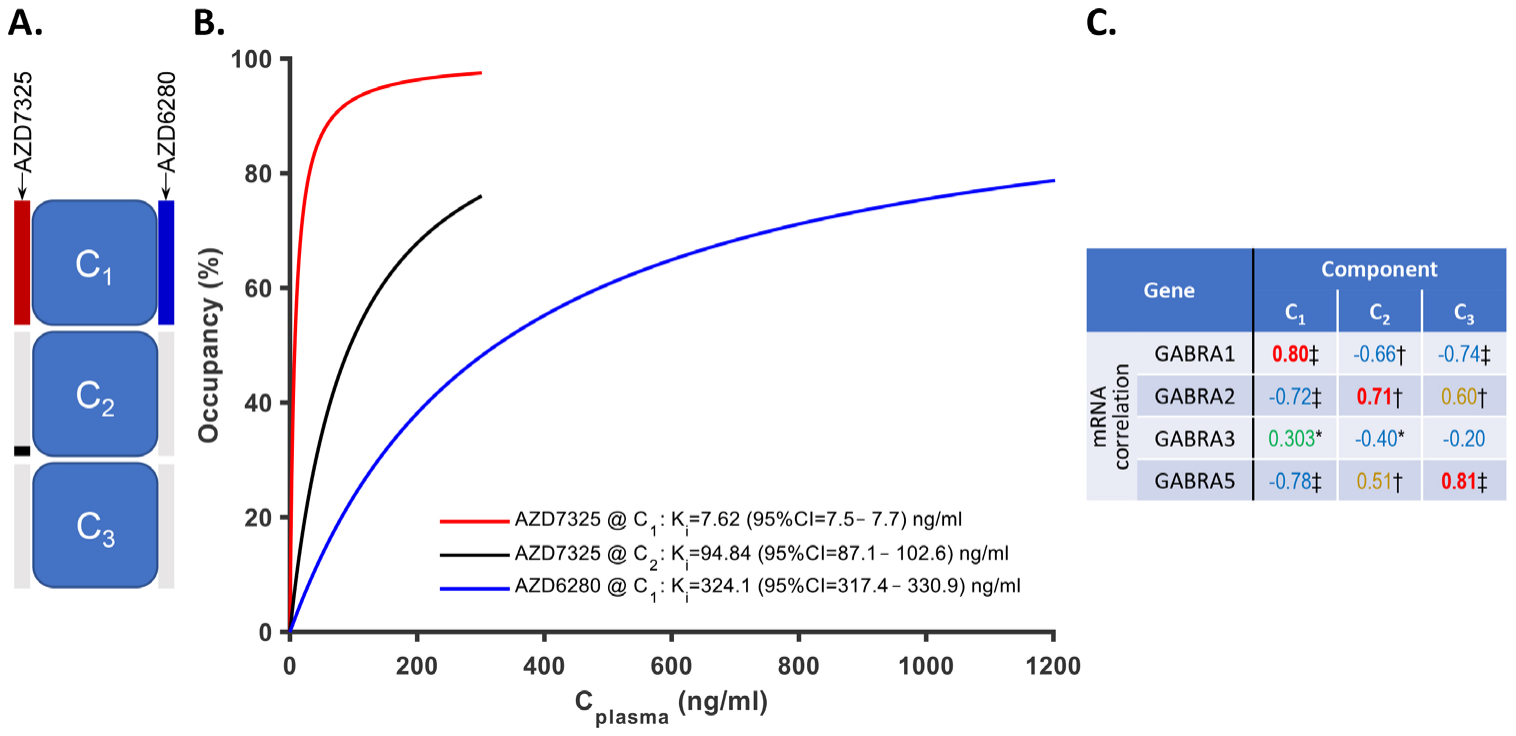
(A) Schematic view of model configuration M3c. The model includes three components of [^11^C]flumazenil binding (C_1_–C_3_). AZD7325 can detectably occupy components C_1_and C_2_, whereas AZD6280 occupies only component C_1_. The bars on the left and right sides of each component box in the schematic view of the model indicate the binding of AZD7325 and AZD6280, respectively, to the given component. The colored part of the bars indicates the detectable presence and relative magnitude of the binding affinity (1/K_i_) of each drug to the given component with colors matching those used in the curvilinear plot. (B) The model-fitted curvilinear relationships between plasma drug exposure and component-wise occupancy for AZD7325 and AZD6280, partial agonists at the BZ site on the GABA_A_R. (C) The table of correlation coefficients between fractional gene expression and model-predicted component contribution across 62 brain regions according to the Allen Human Reference Atlas (AHRA). For each component, the largest correlation coefficient is shown in bold. The color of the correlation coefficient corresponds to the size of the correlation as described inSection 2. Symbol next to the correlation coefficient indicates the level of the corresponding FDR-corrected p-value:^‡^<1 × 10^-10^≤^†^< 1 × 10^-3^≤ * < 0.05.

The M3c model has three binding components (Fig. 3A). AZD7325 was able to occupy binding components 1 and 2, whereas AZD6280 occupied only component 1. For AZD7325, there was a 12-fold difference between the 2 component-specific affinity parameters (K_i_) with the higher affinity corresponding to component 1 (Fig. 3B). The difference was statistically significant, at the α = 5% level, as indicated by the narrow, non-overlapping 95% confidence intervals of the 2 K_i_estimates. Cortical surface and non-cortical volumetric maps of component-specific contributions for the selected model are available online athttps://doi.org/10.12751/g-node.83wfme.

### 3.2 Regional pattern of [ ^11^ C]flumazenil binding

[^11^C]flumazenil binding (BP_ND_) across cortex, which is the foundational data for the presented analysis, was visualized on a left cortical flat map with demarcations of the Desikan–Killiany atlas cortical parcels (left panels inFig. 4A, B; see alsoSupplementary Figs. S2with demarcations according to the AHRA cortical parcels). Cortical binding is also shown in more detail for both hemispheres on 3D inflated cortical surfaces in the left panels ofSupplementary Figures S3 and S4. Subcortical binding is shown in 3D volume rendering in the right panel ofFigure 4A(subcortical segmentation shown in the right panel ofFig. 4B) and on a montage of 2D cross sections with segment borders according to FreeSurfer’s subcortical segmentation in the right panels ofSupplementary Figures S3 and S4, respectively. A tabulation of regional mean [^11^C]flumazenil binding values is available inSupplementary File 4.

**Fig. 4.**
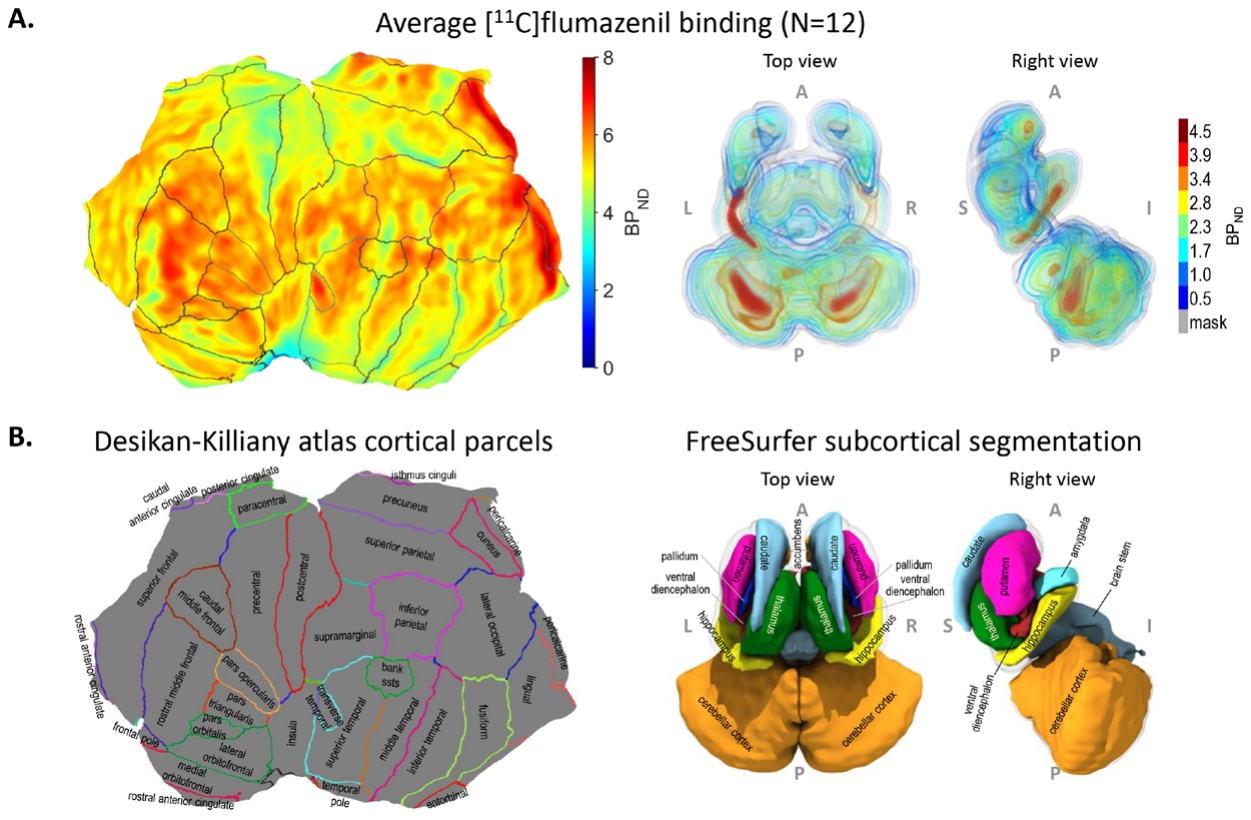
(A) [^11^C]flumazenil binding in the left cerebral cortex displayed on flattened cortical surface on the left. Top and right-side view of volume renderings of [^11^C]flumazenil binding in valid subcortical voxels (gray surface showing the mask of valid subcortical voxels) on the right. (B) Anatomical parcellation of the cortical surface according to the Desikan–Killiany atlas in FreeSurfer with names of the cortical parcels on the left. The boundary lines in A (left panel) were transferred from B (left panel). 3D surface rendering of FreeSurfer’s segmentation of subcortical voxels with names of the structures on the right.

As seen on the surface maps, the cortical binding potential (BP_ND_) was typically above 4.0 with highest values for the occipital cortex (Fig. 4A,Supplementary Figs. S2 and S3). Subcortical values were generally lower with voxel-wise values reaching 4.0 or higher only in hippocampus and cerebellar cortex (Fig. 4A,Supplementary Fig. S3).

**Fig. 5.**
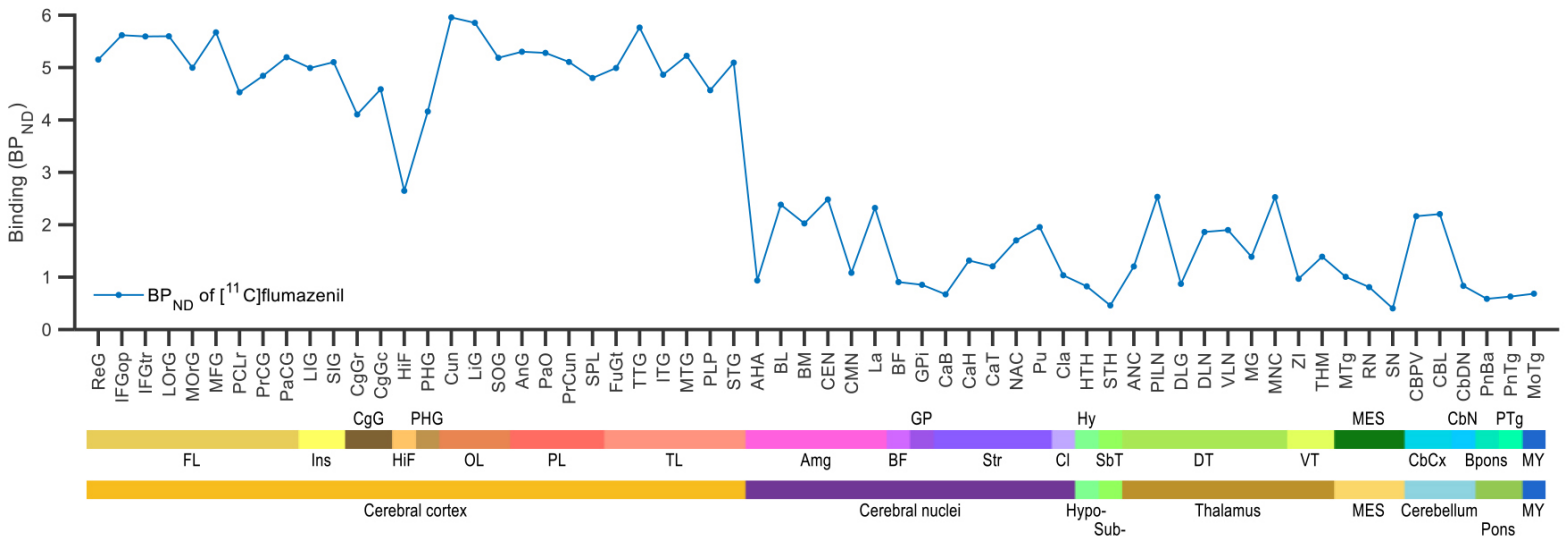
Inter-individual average [^11^C]flumazenil binding (N = 12) in substructures defined according to the Allen Human Reference Atlas (AHRA). The substructures are ordered in rostro-caudal direction. Major brain region and structural classification according to the AHRA is indicated at the bottom with abbreviations and colored stripes. FL: frontal lobe, Ins: insula, CgG: cingulate gyrus, HiF: hippocampal formation, PHG: parahippocampal gyrus, OL: occipital lobe, PL: Parietal lobe, TL: temporal lobe, Amg: amygdala, BF: basal forebrain, GP: globus pallidus, Str: striatum, Cl: claustrum, Hy: hypothalamus, SbT: subthalamus, DT: dorsal thalamus, VT: ventral thalamus, MES: mesencephalon, CbCx: cerebellar cortex, CbN: cerebellar nuclei, Bpons: basal part of the pons, PTg: pontine tegmentum, MY: myelencephalon. See substructure names and abbreviations in columns 7 and 8 of the spreadsheet inSupplementary File 2, respectively.

Furthermore, [^11^C]flumazenil BP_ND_values were plotted for the 62 anatomical regions defined by the anatomically more detailed AHRA that were used for correlation with gene expression (Fig. 5, for a plot for all 95 AHRA substructures with binding data, seeSupplementary Fig. S5). The highest region-specific average cortical value was 6.0 BP_ND_and the cortical binding was below 4.0 only in the hippocampal formation (HiF) and limen insula (LI, seeSupplemental Fig. S5). Regional subcortical [^11^C]flumazenil BP_ND_was at or below 2.5 (Fig. 5).

### 3.3 Brain pattern of component-specific fractional contributions

**Fig. 6.**
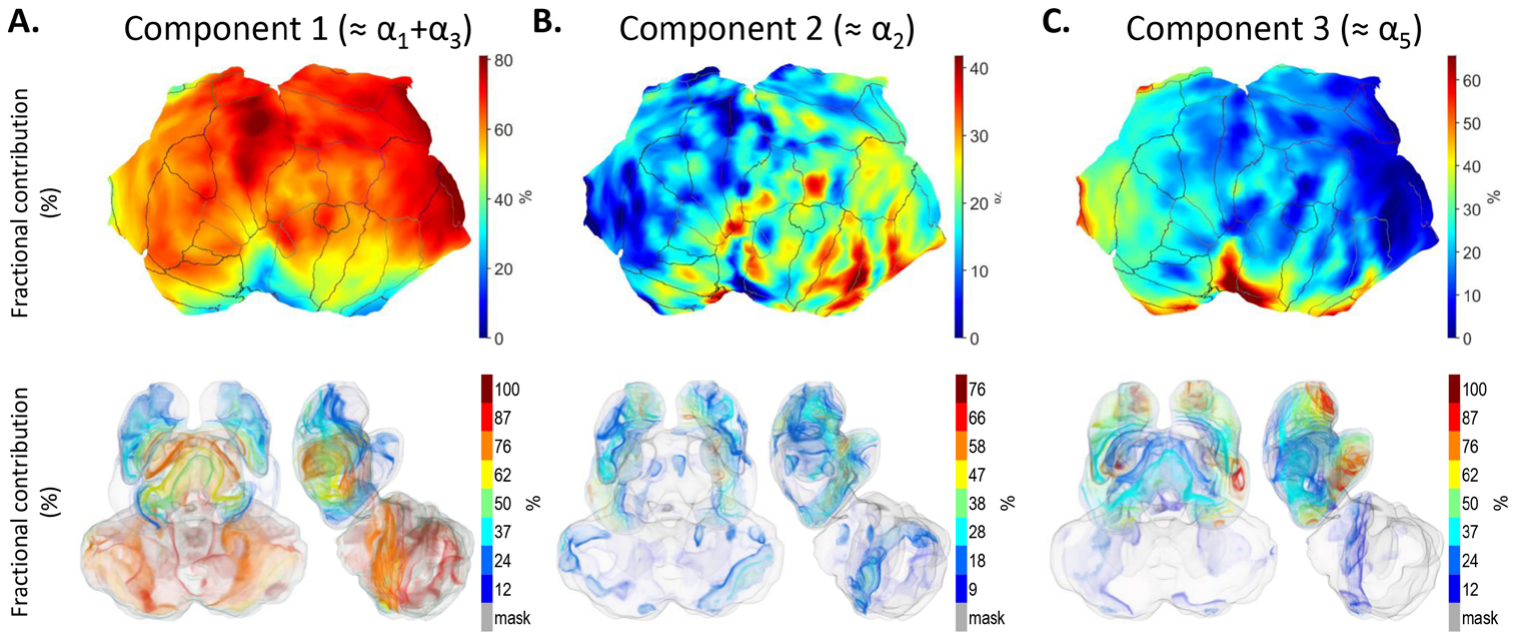
(A–C) Component-specific fractional (%) contribution to overall [^11^C]flumazenil binding in the left cerebral cortex displayed on flattened cortical surface (top panels). Anatomical parcellation of the cortical surface according to the Desikan–Killiany atlas in FreeSurfer is indicated with boundary lines. Volume rendering of component-specific fractional contribution (%) to overall [^11^C]flumazenil binding in valid subcortical voxels, gray surface shows the mask of valid subcortical voxels (bottom panels). Color bars indicate approximate values for the corresponding translucent isosurfaces. The tentative correspondence of the model-specific binding components to α-subunit expression, based on similarity of their inter-regional pattern to that of gene expression data, is shown in parentheses for each component.

The component-specific fractional (%) contributions to binding across the brain were visualized in a similar manner to overall [^11^C]flumazenil binding (Figs. 6and7;Supplementary Figs. S6to S10). Taken as a whole, the component-specific patterns were distinct and did not line up with each other or with the pattern for overall [^11^C]flumazenil binding. Regional mean component-specific fractional contributions are available inSupplementary File 4.

**Fig. 7.**
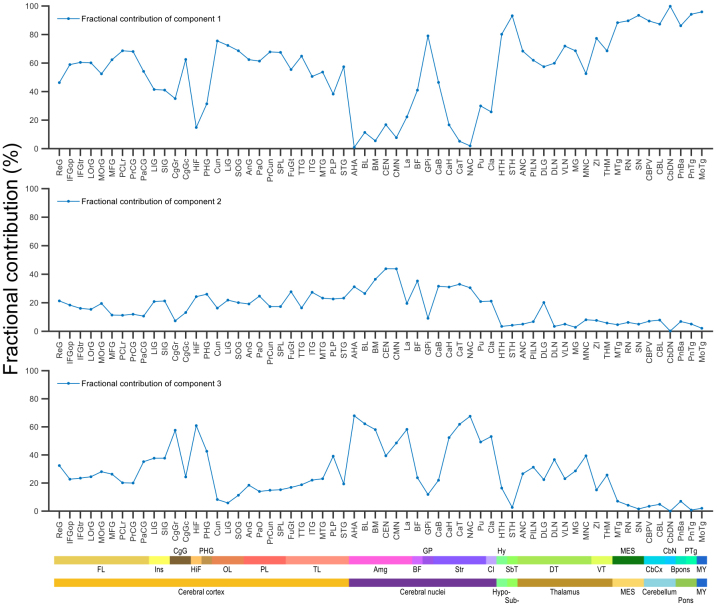
Fractional (%) contribution of the three model-derived components (C_1_–C_3_) to overall [^11^C]flumazenil binding in substructures defined according AHRA. The substructures are ordered in the rostro-caudal direction. Major brain region and structural classification according to the AHRA are indicated at the bottom with abbreviations and colored stripes. FL: frontal lobe, Ins: insula, CgG: cingulate gyrus, HiF: hippocampal formation, PHG: parahippocampal gyrus, OL: occipital lobe, PL: parietal lobe, TL: temporal lobe, Amg: amygdala, BF: basal forebrain, GP: globus pallidus, Str: striatum, Cl: claustrum, Hy: hypothalamus, SbT: subthalamus, DT: dorsal thalamus, VT: ventral thalamus, MES: mesencephalon, CbCx: cerebellar cortex, CbN: cerebellar nuclei, Bpons: basal part of the pons, PTg: pontine tegmentum, MY: myelencephalon. See substructure names and abbreviations in columns 7 and 8 of the spreadsheet inSupplementary File 2, respectively.

According to the cortical maps, the fractional contribution of component 1, which could be occupied by both drug candidates, was typically above 60% across the cortex with values up to ~80% in the primary somatosensory/motor cortex and in the pericalcarine cortex (upper panel inFig. 6A,Supplementary Fig. S6A, left panel inSupplementary Fig. S7). On the contrary, the contribution of component 1 was low in the limbic, cingulate, and insular cortices. In most subcortical cerebral nuclei, the regional distribution of component 1 was low (<20%) to moderate (20–50%), whereas it was high (~80%) in globus pallidus (lower panel inFig. 6A, right panel inSupplementary Fig. S7, regional plot inFig. 7,Supplementary Fig. S10). The contribution in thalamus was similar to cortical levels (~60%), but reached above 80% in sub- and hypothalamus, mesencephalon, cerebellar cortex, and myelencephalon.

As visualized in the cortical surface maps, the fractional contribution of component 2, which was only displaceable by AZD7325, and not by AZD6280, was overall lower than component 1, and displayed a gradient of increasingly higher levels across cortical regions in an anterior-to-posterior and superior-to-inferior direction (upper panel inFig. 6B,Supplementary Fig. S6B, left panel inSupplementary Fig. S8). The highest contributions, with up to ~40%, were observed in the temporal, insular, and parietal cortices. The contribution of component 2 was slightly higher in most cerebral nuclei than in cortex, where its contribution was at or below 40% at the voxel level (Fig. 6B,Supplementary Fig. S8), below 20% regionally (Fig. 7,Supplemental Fig. S10). In the diencephalon, mesencephalon, and cerebellum, the contribution was also typically low (below 20% voxel-wise, below 10% regionally) with higher level only in the dorsal lateral geniculate nucleus of thalamus (lower panel inFigs. 6Band7, right panel inSupplementary Figs. S8 and S10).

Component 3, which was not displaceable by either of the two drugs, had high fractional contribution in sharply demarcated areas in the limbic, cingulate, and insular cortices (upper panel inFig. 6C,Supplementary Fig. S6C, left panel inSupplementary Fig. S9). Specifically, the contribution was up to ~65% in these areas, whereas it was below 40% in the other parts of the cortex. The contribution of component 3 in subcortical nuclei was generally similar to the highest cortical levels with the regional average reaching almost 70% (voxel-wise approaching 100%) in amygdalohippocampal area and nucleus accumbens. However, values in globus pallidus and caudate body were low (below 50% at the voxel level, below 25% regionally) (lower panel inFig. 6C,Fig. 7, right panel inSupplementary Figs. S9 and S10). The contribution in the thalamus was about 35%, whereas it was negligible (below 15% voxel-wise, below 10% regionally) in subthalamus, mesencephalon, and below.

### 3.4 Brain pattern of component-specific absolute contributions

The fractional contributions were multiplied by the intersubject mean [^11^C]flumazenil binding to obtain the absolute component-specific contributions expressed as BP_ND_. The cortical surface view, subcortical volume and section views, and regional summary of the absolute contributions are shown inFigs. 8and9,Supplementary Figs. S11 to S15). Regional mean absolute contributions are available inSupplementary File 4.

**Fig. 8.**
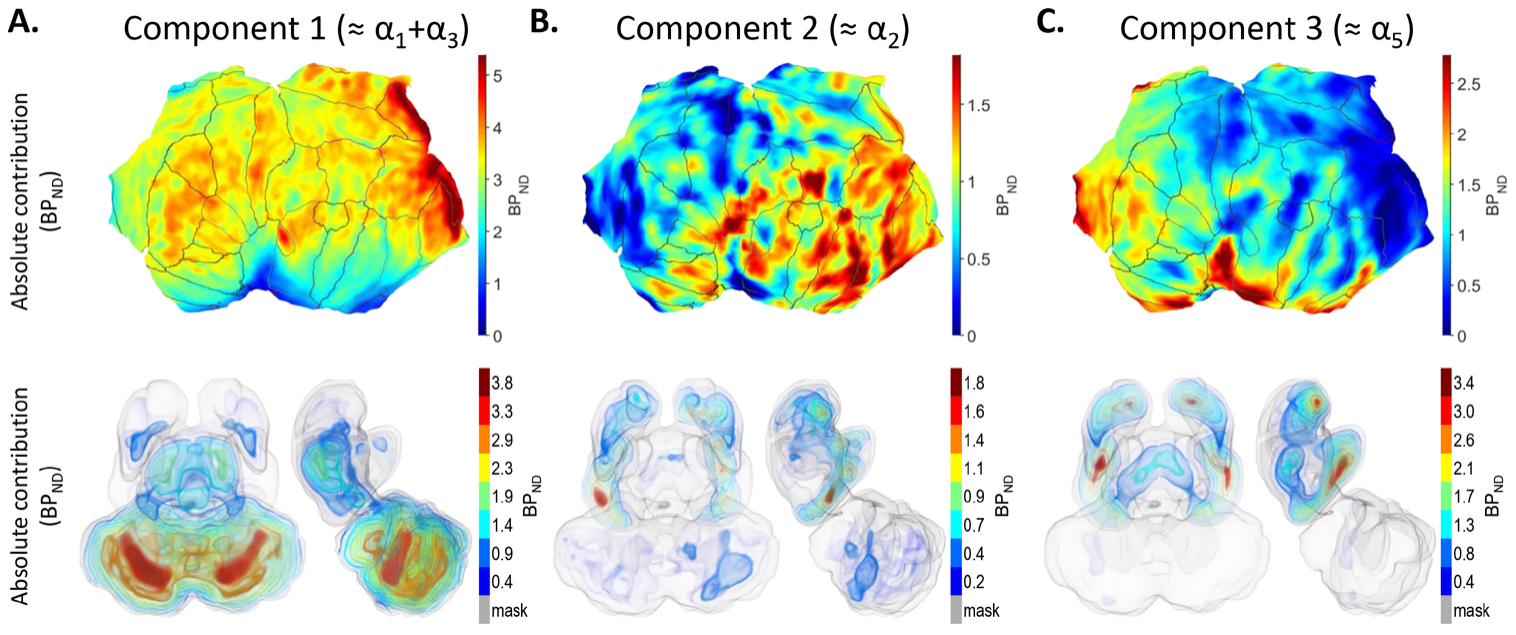
(A–C) Component-specific absolute (BP_ND_) contribution to overall [^11^C]flumazenil binding in the left cerebral cortex displayed on flattened cortical surface (top panels). Anatomical parcellation of the cortical surface according to the Desikan–Killiany atlas in FreeSurfer is indicated with boundary lines. Volume rendering of component-specific absolute (BP_ND_) contribution to overall [^11^C]flumazenil binding in valid subcortical voxels, gray surface shows the mask of valid subcortical voxels (bottom panels). Color bars indicate approximate values for the corresponding translucent isosurfaces. The tentative correspondence of the model-specific binding components to α-subunit expression, based on similarity of their inter-regional pattern to that of gene expression data, is shown in parentheses for each component.

**Fig. 9.**
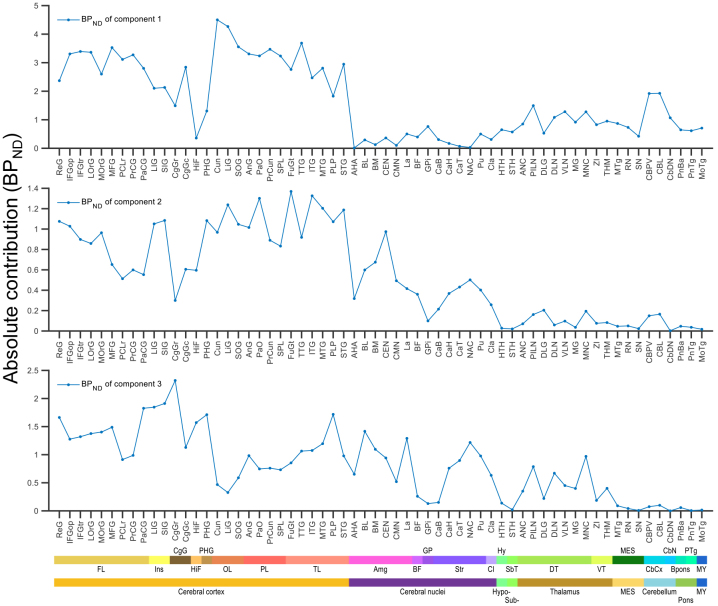
Absolute (BP_ND_) contribution of the three model-derived components (C_1_–C_3_) to overall [^11^C]flumazenil binding in substructures defined according AHRA. The substructures are ordered in the rostro-caudal direction. Major brain region and structural classification according to the AHRA are indicated at the bottom with abbreviations and colored stripes. FL: frontal lobe, Ins: insula, CgG: cingulate gyrus, HiF: hippocampal formation, PHG: parahippocampal gyrus, OL: occipital lobe, PL: parietal lobe, TL: temporal lobe, Amg: amygdala, BF: basal forebrain, GP: globus pallidus, Str: striatum, Cl: claustrum, Hy: hypothalamus, SbT: subthalamus, DT: dorsal thalamus, VT: ventral thalamus, MES: mesencephalon, CbCx: cerebellar cortex, CbN: cerebellar nuclei, Bpons: basal part of the pons, PTg: pontine tegmentum, MY: myelencephalon. See substructure names and abbreviations in columns 7 and 8 of the spreadsheet inSupplementary File 2, respectively.

As seen on the cortical maps, the absolute cortical contribution of component 1 was highest in the pericalcarine cortex, followed by the transverse temporal cortex (upper panel inFig. 8A,Supplementary Fig. S11A, left panel inSupplementary Fig. S12). The absolute contribution of component 1 was lowest in the limbic, cingulate, and insular cortices. The absolute contribution of component 1 in most subcortical regions was below typical cortical values (BP_ND_values of 0.0–2.0 vs. ~3.0, respectively), despite the previously indicated high fractional contribution in several subcortical structures (Fig. 9,Supplementary Fig. S15). However, higher BP_ND_values (up to ~3.8 on the voxel level) were observed in parts of the cerebellar cortex (CBL) and the dentate nucleus (CbDN) (lower panel inFigs. 8Aand9, right panel inSupplementary Figs. S12 and S15).

The cortical values for the absolute contribution of component 2 covered a range from near 0 to ~1.8 on the vertex level with gradually higher values in an anterior-posterior and superior-inferior direction (lower panel inFig. 8B,Supplementary Fig. S11B, left panel inSupplementary Fig. S13). As seen on the plot of regional values, cortical surface and subcortical views, the absolute (BP_ND_) contribution for component 2 was generally different between telencephalon (cortex with values up to ~1.8 in distinct patches; cerebral nuclei) and the remaining brain regions (thalamus and below), with voxel-wise values below 0.5, regional values below 0.2 (Figs. 8Band9,Supplementary Figs. S11, S13, and S15).

The absolute contribution of component 3 in cortex ranged between 0.0 and ~3.5 in such a way that values above ~2.0 were confined to sharply demarked cortical areas in the limbic, cingulate, and insular cortices (upper panel inFig. 8C,Supplementary Fig. S11C, left panel inSupplementary Fig. S14). The plot of regional values and subcortical views showed that the absolute contribution of component 3 was detectable in prosencephalon (thalamus and above) and negligible in the rest of the brain (lower panel inFigs. 8Cand9, right panel inSupplementary Figs. S14 and S15).

### 3.5 Correlations of component-specific contributions and α-subunit gene expression

**Fig. 10.**
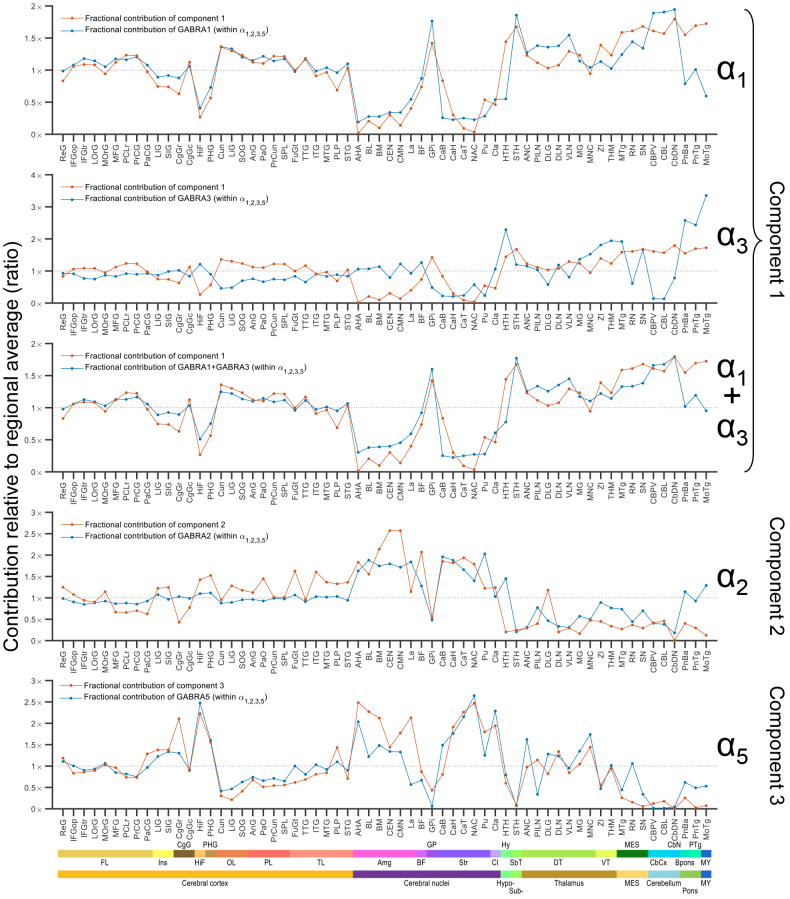
Estimated regional fractional (%) contribution of the three model-derived components (C_1_–C_3_) to overall [^11^C]flumazenil binding in comparison with published gene expression data (Hawrylycz et al., 2012) in substructures defined according to AHRA. The substructures are ordered in the rostro-caudal direction. Gene expression values correspond to the proportion of specific α-subunit mRNA expression in percentage of the summed expression of the α_1_-, α_2_-, α_3_-, and α_5_-subunits. For each component, the charts show relevant comparisons selected based on considering all possible component–gene correlations. In detail, in case of component 1, the comparison with the fractional expression of GABRA1 (R = 0.80, R^2^= 0.64, FDR-corrected p-value = 1 × 10^-14^), GABRA3 (R = 0.303, R^2^= 0.09, FDR-corrected p-value = 0.017), and with the summed fractional expression of GABRA1 and GABRA3 (R = 0.87, R^2^= 0.75, FDR-corrected p-value = 5 × 10^-19^) are shown. In the case of component 2, the comparison with the fractional expression of GABRA2 (R = 0.71, R^2^= 0.51, FDR-corrected p-value = 2 × 10^-10^) is shown. And in the case of component 3, the comparison with the fractional expression of GABRA5 (R = 0.81, R^2^= 0.65, FDR-corrected p-value = 1 × 10^-14^) is shown. To aid visual comparison between the inter-regional patterns, the gene expression and component-specific fractional contribution data were normalized to the average value across brain regions (note that correlation coefficients and p-values are not affected by such a normalization). Major brain region and structural classification according to the AHRA is indicated at the bottom with abbreviations and colored stripes. FL: frontal lobe, Ins: insula, CgG: cingulate gyrus, HiF: hippocampal formation, PHG: parahippocampal gyrus, OL: occipital lobe, PL: parietal lobe, TL: temporal lobe, Amg: amygdala, BF: basal forebrain, GP: globus pallidus, Str: striatum, Cl: claustrum, Hy: hypothalamus, SbT: subthalamus, DT: dorsal thalamus, VT: ventral thalamus, MES: mesencephalon, CbCx: cerebellar cortex, CbN: cerebellar nuclei, Bpons: basal part of the pons, PTg: pontine tegmentum, MY: myelencephalon. See substructure names and abbreviations in columns 7 and 8 of the spreadsheet inSupplementary File 2, respectively.

For biological interpretation and validation, the fractional component contributions for the AHRA substructures were correlated with published gene expression data (Hawrylycz et al., 2012) expressed in fractional units (seeSupplementary File 5with merged gene expression, component contribution, and overall [^11^C]flumazenil binding data). Correlation coefficients are shown inFigure 3C. For visual comparison, the gene expression and component contribution data were plotted for selected gene and component pairs (Fig. 10). To aid visual evaluation, the regional values were normalized to the average value across brain regions, whereas un-normalized values are shown inSupplementary Figure S16.

The contribution of component 1, which was displaced by both drugs, agreed best with the expression of the α_1_-subunit gene (statistically significant positive correlation, R = 0.80 [cross-validation set R = 0.80 ± 0.011, 0.78–0.83], FDR-corrected p-value = 1 × 10^-14^) as compared with the gene expression of the other flumazenil recognized α-subunits (Fig. 3C). Visual inspection of the corresponding plots also suggested agreement (topmost chart inFig. 10andSupplementary Fig. S16). Based on further considerations, the comparisons between component 1 and GABRA3 (R = 0.303 [cross-validation set R = 0.304 ± 0.011, 0.28–0.32], FDR-corrected p-value = 0.017) and component 1 and the sum of GABRA1 and GABRA3 (R = 0.87 [cross-validation set R = 0.87 ± 0.009, 0.85–0.89], FDR-corrected p-value = 5 × 10^-19^) were also plotted (Fig. 10,Supplementary Fig. S16).

The regional pattern of the contribution of component 2 correlated most strongly with α_2_- subunit gene expression (R = 0.71 [cross-validation set R = 0.66 ± 0.082, 0.43–0.77], FDR-corrected p-value = 2 × 10^-10^) and the contribution of component 3 correlated with α_5_-subunit gene expression (R = 0.81 [cross-validation set R = 0.80 ± 0.011, 0.77–0.81], FDR-corrected p-value = 1 × 10^-14^). In both cases, there was visual similarity of the compared patterns (Fig. 10,Supplementary Fig. S16).

## 4 Discussion

In the present image analysis, we took advantage of two previous PET studies in which the occupancy of [^11^C]flumazenil binding to GABA_A_R had been estimated in the human brain after administration of two drug candidates with different binding characteristics at α-subunits (Jucaite et al., 2017). An important observation was that the drug candidates induced different regional patterns of drug occupancy in the brain. Taking advantage of the different occupancy patterns, the present extended image analysis revealed three components of [^11^C]flumazenil binding. The α-subunits (α_1_, α_2_, α_3_, α_5_) corresponding to each component were putatively identified using GABA_A_R subunit gene expression data (Hawrylycz et al., 2012). The analysis confirms that the pharmaceutical industry has been able to generate GABA_A_R modulators that are partly selective at the α-subunit and encourages the development of even more selective compounds with potential for improved drug treatments of neurological and psychiatric disorders.

### 4.1 Further points regarding the comparison with gene expression data

The comparison of model fitting results with gene expression data focused on correlating the component-specific contributions with gene expression of the α-subunit genes GABRA1, GABRA2, GABRA3, and GABRA5, respectively.

The contribution of component 1 was positively correlated with α_1_- and α_3_-subunit gene expression with a high-degree positive correlation with the summed gene expression of these two subunits (Fig. 10,Supplementary Fig. S16). Given the lack of a positive, significant correlation between the gene expression of these subunits (R = -0.12, FDR-corrected p-value = 0.59), a likely interpretation is that component 1 incorporates both α_1_- and α_3_-subunit distribution. At the same time, among the flumazenil detected α-subunits, the gene expression of the α_3_-subunit is generally low (Supplementary Fig. S16). Furthermore, the low α_3_expression levels may explain why disentangling a purely α_3_-related component of [^11^C]flumazenil binding was not successful.

The contribution of component 2 was positively correlated with α_2_and, to a lower degree, with α_5_-subunit gene expression (Figs. 3Cand10,Supplementary Fig. S16). Importantly, the gene expression of these two subunits has previously been shown to correlate (Sequeira et al., 2019), which is consistent with our analysis (R = 0.43, FDR-corrected p-value = 6 × 10^-4^). Taken together with the fact that previous*in vitro*binding assays of the two drugs indicated a lack of substantial affinity to α_5_-subunit-containing GABA_A_Rs, the most plausible interpretation is that component 2 preferentially represents α_2_-subunit expression.

Conversely, component 3’s contribution was positively correlated with α_5_and, to a lower degree, with α_2_-subunit gene expression (Figs. 3Cand10,Supplementary Fig. S16). Considering that component 3 represents flumazenil binding not displaced by either drug, component 3 appears to be more directly related to α_5_-subunit expression. As a further confirmation, the pattern here described for component 3 shows a strong similarity to prior published results obtained using the α_5_-selective radioligand [^11^C]Ro15-4513 (Myers et al., 2017).

The interpretations discussed above were based on correlations of fractional expression values. It is worth noting that correlations performed on*absolute*component contributions (BP_ND_) versus*absolute*gene expression data overall concurred with the findings based on fractional values, albeit with numerically lower degree, statistically less significant associations (data inSupplementary File 5).

Previous publications comparing [^11^C]flumazenil binding profiles in brain with gene expression of various GABA_A_R α- (and other) subunits tended to find only weak-to-moderate associations, the latter in case of GABRA1 (Hansen et al., 2022;Nørgaard et al., 2021). This is expected since [^11^C]flumazenil comprises the summed signal of several subtypes of which the α_1_-dependent signal is the dominant one. The relatively smaller contributions of the other subunits are likely to influence the sum only to a lesser degree. The correlations between [^11^C]flumazenil binding and the expression of relevant subunit genes (i.e., GABRA1, GABRA2, GABRA3, GABRA5) in our data concur with previous findings (Supplementary Table S2, data inSupplementary File 5). However, our analysis went further. By dissecting the [^11^C]flumazenil signal, we were able to reveal strong correlations between its components and α-subunit gene expression.

Furthermore, in addition to the component-specific comparisons, the overall [^11^C]flumazenil binding was also compared with the summed absolute expression of the relevant α-subunit genes (i.e., GABRA1 + GABRA2 + GABRA3 + GABRA5,Supplementary Fig. S17, data in Supplementary File 5). While visual inspection suggests some similarity and the correlation was of high positive degree (R = 0.72, p-value = 2.5 × 10^-10^), the aforementioned level difference between cortical and non-cortical regions in [^11^C]flumazenil binding (Figs. 4and5) was less pronounced in the case of gene expression (Supplementary Fig. S17). Of interest is that a more pronounced cortical-to-non-cortical difference in the case of radioligand binding versus gene expression has been demonstrated previously (see Fig. 3A inNørgaard et al., 2021). It cannot be excluded that differences in the processing of cortical and non-cortical structures may contribute to this discrepancy. Yet the striking regional differences in [^11^C]flumazenil binding are conspicuous even in unprocessed PET radioactivity images or parametric images (seeFig. 1), as well as shown by*in vitro*autoradiography (Braestrup et al., 1977;Nørgaard et al., 2021;Persson et al., 1991). Future work is needed to elucidate the nature of this discrepancy between gene and protein expression.

### 4.2 Methodological considerations

The present assessment relied on the application of combined volume and surface (CVS) registration of individual imaging data to a common template space (MNI152). The traditional approach is to apply the CVS-registration parameters to acquired dynamic PET measurements. The novel approach used in the present analysis was to apply the parameters directly to parametric images of [^11^C]flumazenil binding. The parametric images were obtained using a previously described wavelet-aided approach which allows handling of noise in an optimal manner and yields images suitable for assessing binding at high resolution (Cselényi et al., 2006;Turkheimer et al., 2003). Accordingly, the resulting component maps provided detailed information about putative GABA_A_R α-subunit distribution throughout the brain.

A fundamental assumption of the analysis was that [^11^C]flumazenil binding and the expression patterns of the various GABA_A_R subunits are stable across subjects. The baseline [^11^C]flumazenil binding was similar across subjects: the coefficient of variation for the 12 subjects was 23% on average for each volume and surface element. Moreover, the average correlation coefficient in elementwise baseline binding across every pair of subjects (66 pairs for N = 12), that is, the so-called differential stability, was 0.91 (Hansen et al., 2022). Furthermore, strong positive correlation has previously been reported between*post mortem*receptor densities with autoradiography, and regional [^11^C]flumazenil binding in data obtained in different subjects (Nørgaard et al., 2021). In the same report, the corresponding regression was then used to estimate benzodiazepine receptor (BZR) density (B_max_) values within Desikan–Killiany cortical parcels as well as FreeSurfer subcortical segments (Nørgaard et al., 2021). We compared the inter-subject mean regional [^11^C]flumazenil BP_ND_s in the present study with these published B_max_values. The observed correlation had a very high positive degree and was highly significant (R = 0.95, p-value = 3 × 10^-24^, see also the regression plot inSupplementary Fig. S18, tabulated data in Supplementary File 6). Finally, strong, significant correlations between component contribution and gene expression were observed in the validation part of the present study, as indicated above. Taken together, the strong agreements across multiple domains and studies lend support to the remarkable inter-subject stability of the GABA_A_R system regarding subunit gene and protein expression.

Worth noting is that the strong correlations between component contributions and gene expression also support,*post hoc*, the validity of the first assumed key condition for our analysis, that is, that occupancy at a given α-subunit-containing GABA_A_R subtype is the same across brain regions for a given drug and dose. This conclusion follows, since a major violation of this condition would by itself preclude the possibility to achieve high correlations of the regional component contribution patterns with an independent measure on regional subunit levels such as gene expression.

After selection and application of model M3c for the derivation of components, all model variants were reevaluated by calculation of the correlation between component contribution and gene expression. Interestingly, besides the two-component M2a model, the selected M3c model was the only variant that had highly positive correlation (R > 0.70) with gene expression data for each of its components (see insert table inSupplementary Fig. S1). Note that the two models, M3d and M4d, which were deemed to likely overfit the data despite favorable statistical performance, had no high degree positive correlation with gene expression for any of their displaceable components. This reevaluation further strengthens the selection of model M3c as the most appropriate one.

Limitations of the study were the low number of subjects and the lack of head-to-head data for the two drugs. However, relatedly, the strength of the present study was that the same PET system and experimental protocol were employed in all cases. A further limitation could be that pons was used as reference region in the quantification of BP_ND_estimates, instead of using a metabolite-corrected arterial plasma input function (Nørgaard et al., 2021;Odano et al., 2009). However, the original two PET studies (Jucaite et al., 2017) did not include arterial sampling. Earlier studies with [^11^C]flumazenil suggested that pons may have specific binding albeit much lower than gray matter (Braestrup et al., 1977;Persson et al., 1991). Importantly, however, a subsequent study, including a thorough cross-validation of quantification methods using plasma or reference region radioactivity as input, found no detectable specific binding in pons, concluding that using it as the reference regions yields accurate binding estimates (Odano et al., 2009).

The aforementioned strong correlation between our data and previously published results on estimated regional BZR density, obtained from using a measured plasma input function, supports the presently applied methodology (Supplementary Fig. S18). It is important to note that voxels in the pons were not explicitly removed from the analysis. Instead, when identifying valid voxels as described in step 6 of the image processing workflow, most pontine voxels were automatically eliminated due to their below-threshold baseline binding. As shown in the results, the AHRA substructures for the remaining valid basal part of pons and the pontine tegmentum had low average baseline binding. Moreover, encouraged by the strong observed correlation with previously estimated BZR density, we used the equation for the corresponding linear regression (Supplementary Fig. S18) to cast the overall [^11^C]flumazenil binding map to BZR density units from which tentative absolute BZR density (B_max_) contribution maps were also calculated for each component (note that all supplementary tabulated data contain both [^11^C]flumazenil binding and projected BZR density data).

### 4.3 Extended mapping of GABA _A_ α-subunit receptor distribution

GABA_A_Rs containing different α-subunits are known to have differential distribution in rodent brain and have been associated with different functional roles (Fritschy & Mohler, 1995;Rudolph et al., 2001). Knowledge on GABA_A_R subunit distribution in the human brain has primarily emerged from immunohistochemistry studies. Though very detailed, those studies have been limited to selective regions, such as hippocampus, amygdala, subthalamic nucleus, or certain cortical areas (Sacco et al., 2009;Stefanits et al., 2018;Wu et al., 2018). In this perspective, the main contribution of the present study is a neuroanatomically more exhaustive map of putative GABA_A_R α-subunit distribution across all brain regions in the human brain*in vivo*. To facilitate use of these maps for further research, the component contributions, overall flumazenil binding (BP_ND_), and BZR density (B_max_) maps, as well as the AHRA atlas re-formatted as described inSection 2, are made available online athttps://doi.org/10.12751/g-node.83wfme. We see potential application of fractional maps for drug development, informing on the distribution of and balance between α-subunits as specific targets for selective drugs. In addition, the absolute GABA_A_R subunit maps may be of interest for development of selective radioligands and consequently studying brain biochemistry and pathophysiology of CNS disorders.

## 5 Conclusions

The present study extends knowledge on the GABA_A_R system by demonstrating the feasibility of disentangling specific patterns to the GABA_A_Rs complexes containing differentiated α-subtypes*in vivo*in the human brain. Further development of the molecular imaging toolbox could in turn invigorate the search for more optimized subtype-selective GABA_A_R compounds. The success of these efforts would be of benefit in basic research as well as in developing treatment for neurological and psychiatric disorders.

## Supplementary Material

Supplementary Material

## Data Availability

Besides the comprehensive online data and results depository described inSection 4.2, other data used in the manuscript will be made available on request by email to the corresponding author pending approval.

## Supplementary Materials

Supplementary figures, tables, and tabulated data can be found in the online version of the article, athttps://doi.org/10.1162/imag_a_00464. The first supplementary file contains a list and short description of all the supplements.
