# Supplementary material for: Putative mapping of α-subunits in the human brain: A PET study of GABA A receptor binding
Source: Imaging Neurosci (Camb). 2025 Jan 30;3:imag_a_00464. doi: 10.1162/imag_a_00464 (PMC12319990; doi:10.1162/imag_a_00464)
Supplement: Supplementary Material [file imag_a_00464-supp.zip › 1_Supplementary_document.pdf]

Supplementary Materials for:

# Putative mapping of α-subunits in the human brain – a PET study of $GABA_A$ receptor binding

Zsolt Cselényi[1,2] MD, PhD; Aurelija Jucaite[1,2] MD, PhD; Lars Farde[2] MD, PhD

*[1]PET Science Centre, Personalized Medicine and Biosamples, R&D, AstraZeneca, Stockholm, Sweden; [2]PET Centre, Department of Clinical Neuroscience, Centre for Psychiatry Research, Karolinska Institutet, Stockholm, Sweden*

Supplementary files:

1. **(This file)**: contains supplementary file listing.
2. **Mapping_AHRA_ROIs_to_AHBA_substructures.xlsx**: Excel spreadsheet providing the mapping between 3D Allen Human Reference Atlas (AHRA) substructures and regions of interest (ROIs) and Allen Human Brain Atlas (AHBA) substructures (Hawrylycz et al., 2012; Song-Lin Ding, Joshua J. Royall, Susan M. Sunkin, Benjamin A.C. Facer, Phil Lesnar, Amy Bernard, Lydia Ng, Ed S. Lein, 2020).
3. **Supplementary_tables_figures.pptx**: each slide contains a supplementary table or figure. The slide notes contain the table/figure captions and legends.
4. **Component_contribution_ROI_statistics.zip**: contains tabulated data with regional summary of model-predicted component-wise fractional and absolute (binding and density) contributions as well as overall [$^{11}$C]flumazenil binding ($BP_{ND}$) and benzodiazepine-receptor density ($B_{max}$). FreeSurfer's Desikan-Killiany (DK) atlas and the AHRA were applied (Fischl, 2012; Song-Lin Ding, Joshua J. Royall, Susan M. Sunkin, Benjamin A.C. Facer, Phil Lesnar, Amy Bernard, Lydia Ng, Ed S. Lein, 2020). For each ROI the table indicates the corresponding source atlas. The table is available in Excel spreadsheet (.xlsx) and TAB-separated value (.tsv) format. The accompanying .json sidecar file contains metadata describing the table columns.
5. **Data_for_comparison_to_gene_expression.zip**: contains tabulated data with regional gene expression and model-predicted component-wise fractional and absolute (binding and density) contributions as well as overall [$^{11}$C]flumazenil binding ($BP_{ND}$) and benzodiazepine-receptor density ($B_{max}$) values for the matched AHRA substructures (as described in supplement 2). The gene expression data comes from a prior publication (Hawrylycz et al., 2012). The table is available in Excel spreadsheet (.xlsx) and TAB-separated value (.tsv) format. The accompanying .json sidecar file contains metadata describing the table columns.
6. **Data_for_comparison_to_BZR_density.zip**: contains tabulated data with regional estimated $GABA_A$ benzodiazepine-receptor (BZR) density (specifically, $B_{max}$ for BZR) published in a previous study (Nørgaard et al., 2021) and total [$^{11}$C]flumazenil binding ($BP_{ND}$) in the present study according to the DK atlas. The table is available in Excel spreadsheet (.xlsx) and TAB-separated value (.tsv) format. The accompanying .json sidecar file contains metadata describing the table columns.

**References:**

Fischl, B., 2012. FreeSurfer. NeuroImage, 20 YEARS OF fMRI 62, 774–781. https://doi.org/10.1016/j.neuroimage.2012.01.021

Hawrylycz, M.J., Lein, E.S., Guillozet-Bongaarts, A.L., Shen, E.H., Ng, L., Miller, J.A., van de Lagemaat, L.N., Smith, K.A., Ebbert, A., Riley, Z.L., Abajian, C., Beckmann, C.F., Bernard, A., Bertagnolli, D., Boe, A.F., Cartagena, P.M., Chakravarty, M.M., Chapin, M., Chong, J., Dalley, R.A., David Daly, B., Dang, C., Datta, S., Dee, N., Dolbeare, T.A., Faber, V., Feng, D., Fowler, D.R., Goldy, J., Gregor, B.W., Haradon, Z., Haynor, D.R., Hohmann, J.G., Horvath, S., Howard, R.E., Jeromin, A., Jochim, J.M., Kinnunen, M., Lau, C., Lazarz, E.T., Lee, C., Lemon, T.A., Li, L., Li, Y., Morris, J.A., Overly, C.C., Parker, P.D., Parry, S.E., Reding, M., Royall, J.J., Schulkin, J., Sequeira, P.A., Slaughterbeck, C.R., Smith, S.C., Sodt, A.J., Sunkin, S.M., Swanson, B.E., Vawter, M.P., Williams, D., Wohnoutka, P., Zielke, H.R., Geschwind, D.H., Hof, P.R., Smith, S.M., Koch, C., Grant,

S.G.N., Jones, A.R., 2012. An anatomically comprehensive atlas of the adult human brain transcriptome. Nature 489, 391–399. https://doi.org/10.1038/nature11405

Nørgaard, M., Beliveau, V., Ganz, M., Svarer, C., Pinborg, L.H., Keller, S.H., Jensen, P.S., Greve, D.N., Knudsen, G.M., 2021. A high-resolution in vivo atlas of the human brain's benzodiazepine binding site of GABAA receptors. NeuroImage 232, 117878. https://doi.org/10.1016/j.neuroimage.2021.117878

Song-Lin Ding, Joshua J. Royall, Susan M. Sunkin, Benjamin A.C. Facer, Phil Lesnar, Amy Bernard, Lydia Ng, Ed S. Lein, 2020. Allen Human Reference Atlas – 3D, 2020.
