# Supplementary material for: Putative mapping of α-subunits in the human brain: A PET study of GABA A receptor binding
Source: Imaging Neurosci (Camb). 2025 Jan 30;3:imag_a_00464. doi: 10.1162/imag_a_00464 (PMC12319990; doi:10.1162/imag_a_00464)
Supplement: Supplementary Material [file imag_a_00464-supp.zip › 3_Supplementary_tables_figures.pdf]

Supplementary tables & figures for:

Putative mapping of α-subunit-specific components of $GABA_A$ receptor distribution in the human brain – a PET study

Zsolt Cselényi[1,2] MD, PhD; Aurelija Jucaite[1,2] MD, PhD; Lars Farde[2] MD, PhD

[1]PET Science Centre, Personalized Medicine and Biosamples, R&D, AstraZeneca, Stockholm, Sweden; [2]PET Centre, Department of Clinical Neuroscience, Centre for Psychiatry Research, Karolinska Institutet, Stockholm, Sweden

	**M3a**	**M3b**	**M3c**	**M3d**	**M3e**	**M4a**	**M4b**	**M4c**	**M4d**	**M4e**
M2a	**1.03**	**1.03**	**1.71**	**2.42**	0.86	**1.13**	**1.32**	0.97	**1.65**	**1.25**
M3a		-3.52	**4190.45**	**4267.93**		**1.22**	**1.58**	0.91	**2.20**	**1.44**
M3b				**8539.61**		**1.23**	**1.58**	0.91	**2.20**	**1.44**
M3c				**4209.11**		0.59	0.94	0.29	**1.53**	0.80
M3d						-0.03	0.31	-0.32	0.89	0.18
M3e		**1054.97**	**5283.26**	**4832.11**		**1.39**	**1.75**	**1.07**	**2.37**	**1.61**
M4a							**2183.43**	-1899.87	**5912.49**	
M4e						-1306.26	**426.99**	-1592.96	**2271.70**	

Supplementary Table S1. Table of F values (pair-wise one-tailed F-tests, α=0.05) comparing occupancy model variants (see supplementary Fig. S1 for model configurations and names). The less complex model in each comparison is on the left and the more complex one is on the top. If the F-test was not possible (due to equal model complexity) then the corresponding cell is greyed out or the whole row/column is removed if no comparisons are possible for the given model (e.g. M2a as the more complex model or M4b as the less complex model). If the more complex model is preferred in the one-tailed test then the F value has **bold** font and is colored purple, otherwise it has normal font weight and is black.

Gene	R-value	FDR corr. p-value
GABRA1	0.68	1.79E-09
GABRA2	0.36	6.94E-03
GABRA3	0.28	3.03E-02
GABRA5	0.15	2.38E-01

Supplementary Table S2. Correlation between $GABA_A$ receptor α subunit gene expression for the 4 benzodiazepine-sensitive α subunits (non-log2 scale) and $[^{11}C]$flumazenil binding (BP_{ND}) using regional data according in AHRA substructures.

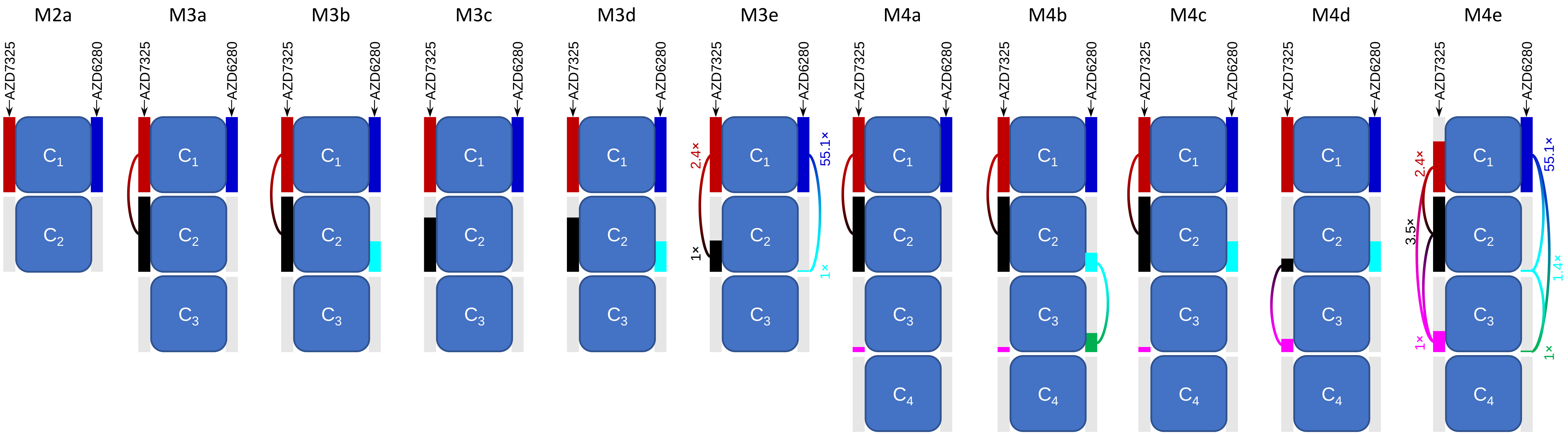

Name	M2a	M3a	M3b	M3c	M3d	M3e	M4a	M4b	M4c	M4d	M4e
K	6471	12940	12941	12941	12942	12940	19410	19411	19411	19411	19409
RESNORM	52825.3	50235.4	50260.8	48659.3	47126.1	50646.4	47192.5	46368.8	47472.3	45026.5	46691
MSE	0.389	0.388	0.388	0.376	0.364	0.391	0.384	0.377	0.390	0.366	0.380
AIC	-128106	-122322	-122248	-126857	-131411	-121162	-118275	-120779	-117432	-124959	-119797
Zeros (%)	6	9	7	9	14	18	24	27	10	17	24
MACV (%)	1.4	1.8	16.0	2.2	6.3	2.2	1.9	4.3	6.2	3.6	3.0

	M2a		M3a			M3b			M3c			M3d			M3e			M4a				M4b				M4c				M4d				M4e			
	C_1	C_2	C_1	C_2	C_3	C_1	C_2	C_3	C_1	C_2	C_3	C_1	C_2	C_3	C_1	C_2	C_3	C_1	C_2	C_3	C_4	C_1	C_2	C_3	C_4	C_1	C_2	C_3	C_4	C_1	C_2	C_3	C_4	C_1	C_2	C_3	C_4
Asymmetry (median|%|)	1	3	3	12	4	6	8	3	2	9	5	22	3	5	53	2	3	3	69	6	8	68	65	2	4	10	19	7	5	42	5	15	4	49	59	3	5
mRNA correlation: GABRA1	**0.80**	-0.80	**0.82**	-0.42	-0.78	**0.81**	-0.01	-0.78	**0.80**	-0.66	-0.74	**0.48**	0.26	-0.80	**0.48**	**0.76**	-0.80	**0.82**	0.01	-0.55	-0.72	**0.45**	**0.301**	**0.37**	-0.80	**0.43**	**0.61**	-0.56	-0.76	**0.47**	**0.36**	-0.55	-0.78	**0.53**	**0.40**	0.21	-0.80
GABRA2	-0.69	**0.69**	-0.73	**0.49**	**0.67**	-0.73	0.12	**0.67**	-0.72	**0.71**	**0.60**	-0.49	-0.13	**0.68**	-0.46	-0.63	**0.69**	-0.73	0.02	**0.57**	**0.56**	-0.45	-0.32	-0.25	**0.68**	-0.28	-0.60	**0.64**	**0.61**	-0.48	-0.24	**0.61**	**0.65**	-0.45	-0.43	-0.09	**0.68**
GABRA3	**0.26**	-0.26	**0.29**	-0.28	-0.25	**0.33**	-0.22	-0.25	**0.303**	-0.40	-0.20	**0.41**	-0.25	-0.20	**0.41**	0.12	-0.24	**0.299**	0.03	-0.30	-0.18	**0.45**	**0.29**	-0.17	-0.22	-0.16	**0.41**	-0.44	-0.20	**0.42**	-0.18	-0.27	-0.20	**0.301**	**0.41**	-0.29	-0.21
GABRA5	-0.80	**0.80**	-0.80	**0.27**	**0.80**	-0.78	-0.09	**0.79**	-0.78	**0.51**	**0.81**	-0.48	-0.27	**0.81**	-0.52	-0.74	**0.80**	-0.80	-0.07	**0.44**	**0.80**	-0.49	-0.28	-0.37	**0.80**	-0.45	-0.57	**0.42**	**0.81**	-0.46	-0.35	**0.37**	**0.81**	-0.60	-0.40	-0.21	**0.80**

Supplementary Figure S1. Schematic view of tested model configurations on top. For details see next page. Table below shows model performance measures (above thick black horizontal separator line, used for model selection) and correlation coefficients for comparisons with gene expression data (below thick black horizontal separator line, used for additional model exploration *after* model selection). Correlation coefficients in bold font indicate the highest value for the given component (colored described on next page).

Explanatory details for Supplementary Figure S1.

Model configurations details:

- The M2a assumed that only the lumped occupancy can be quantified, representing the high-affinity portions of drug binding (C_1).
- In the M3a model AZD7325 had the same affinity for C_1 and C_2, but only C_1 was displaceable by AZD6280.
- In the M3b model AZD7325 had the same affinity for C_1 and C_2, AZD6280 had different affinities for C_1 and C_2 (starting value set to high affinity for C_1).
- In the M3c model AZD7325 had different affinities for C_1 and C_2 (starting value set to high affinity for C_1), but only C_1 was displaceable by AZD6280.
- In the M3d model both AZD7325 and AZD6280 had different affinities for C_1 and C_2 (starting value set to high affinity for C_1 for both drugs).
- In the M3e model AZD7325 had a fixed 2.4:1 ratio of its affinities for C_1 and C_2, respectively (based on *in vitro* affinity figures), AZD6280 had affinities with a fixed 55.1:1 ratio for C_1 and C_2, respectively (based on *in vitro* affinity figures).
- In the M4a model AZD7325 had the same affinity for C_1 and C_2, and a different affinity for C_3 (starting value set to high affinity for $C_{1,2}$). Only C_1 was displaceable by AZD6280.
- In the M4b model AZD7325 had the same affinity for C_1 and C_2, and a different affinity for C_3 (starting value set to high affinity for $C_{1,2}$). AZD6280 had the same affinity for C_2 and C_3, and a different affinity for C_1 (starting value set to high affinity for C_1).
- In the M4c model AZD7325 had the same affinity for C_1 and C_2, and a different affinity for C_3 (starting value set to high affinity for $C_{1,2}$). AZD6280 had different affinities for C_1 and C_2 but could not displace C_3 (starting value set to high affinity for C_1).
- In the M4d model AZD7325 had the same affinity for C_2 and C_3, and a different affinity for C_1 (starting value set to high affinity for C_1). AZD6280 had different affinities for C_1 and C_2 but could not displace C_3 (starting value set to high affinity for C_1).
- In the M4e model AZD7325 had a fixed 2.4:3.6:1 ratio of its affinities for C_1, C_2 and C_3, respectively (based on *in vitro* affinity figures). AZD6280 had affinities with a fixed 55.1:1.4:1 ratio for C_1, C_2 and C_3, respectively (based on *in vitro* affinity figures).

Correlation coefficients were colored according to the following rule of thumb for Interpreting the size of the coefficient:

Size of Correlation	Interpretation	Color
$0.90<R\leq1.00$	Very high positive correlation	**Magenta**
$0.70<R\leq0.90$	High positive correlation	**Red**
$0.50<R\leq0.70$	Moderate positive correlation	**Yellow**
$0.30<R\leq0.50$	Low positive correlation	**Green**
$-1.00<R\leq0.30$	negative or negligible correlation	**Black**

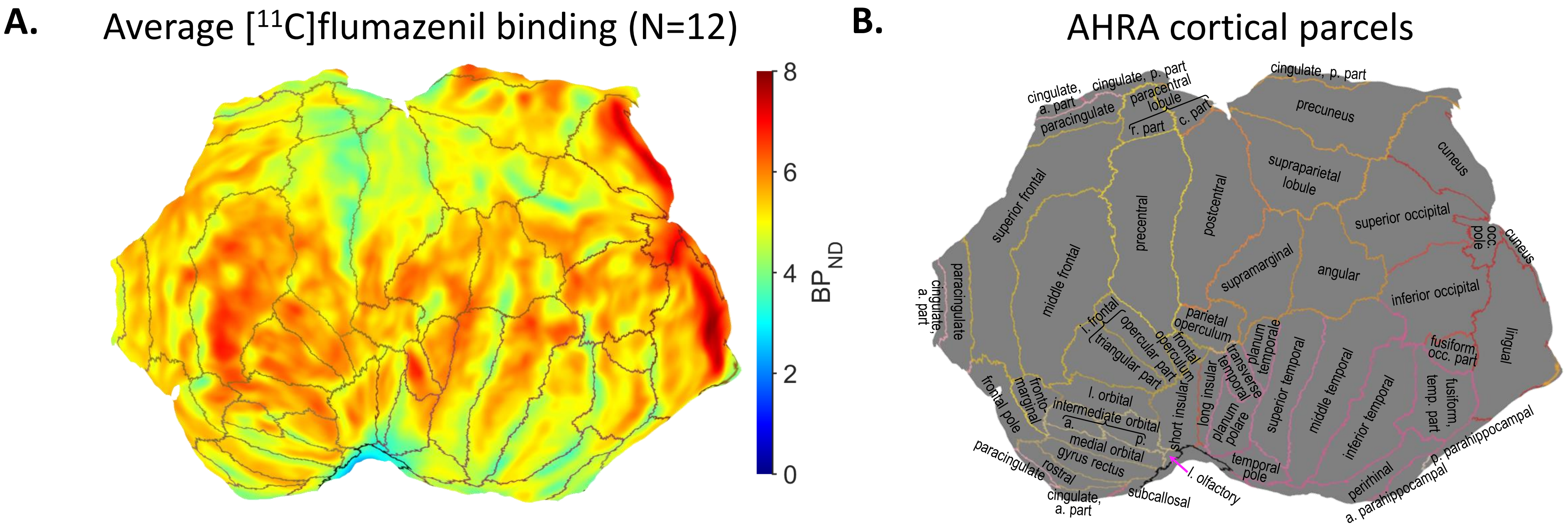

Supplementary Figure S2. (A) [11C]flumazenil binding in the left cerebral cortex displayed on the flattened cortical surface. **(B)** Anatomical parcellation of the cortical surface according to the Allen Human Reference Atlas (AHRA) with names of the cortical parcels. Note that the word "gyrus" or "gyri" (for insular gyri) are omitted from the end of the parcel names, except those designated as "lobule" which is shown. The boundary lines in A were transferred from B.
Abbreviations: a. – anterior; i. – inferior; l. – lateral; occ. – occipital; p. – posterior; temp. – temporal.

Average [11C]flumazenil binding (BP_{ND}, N=12)

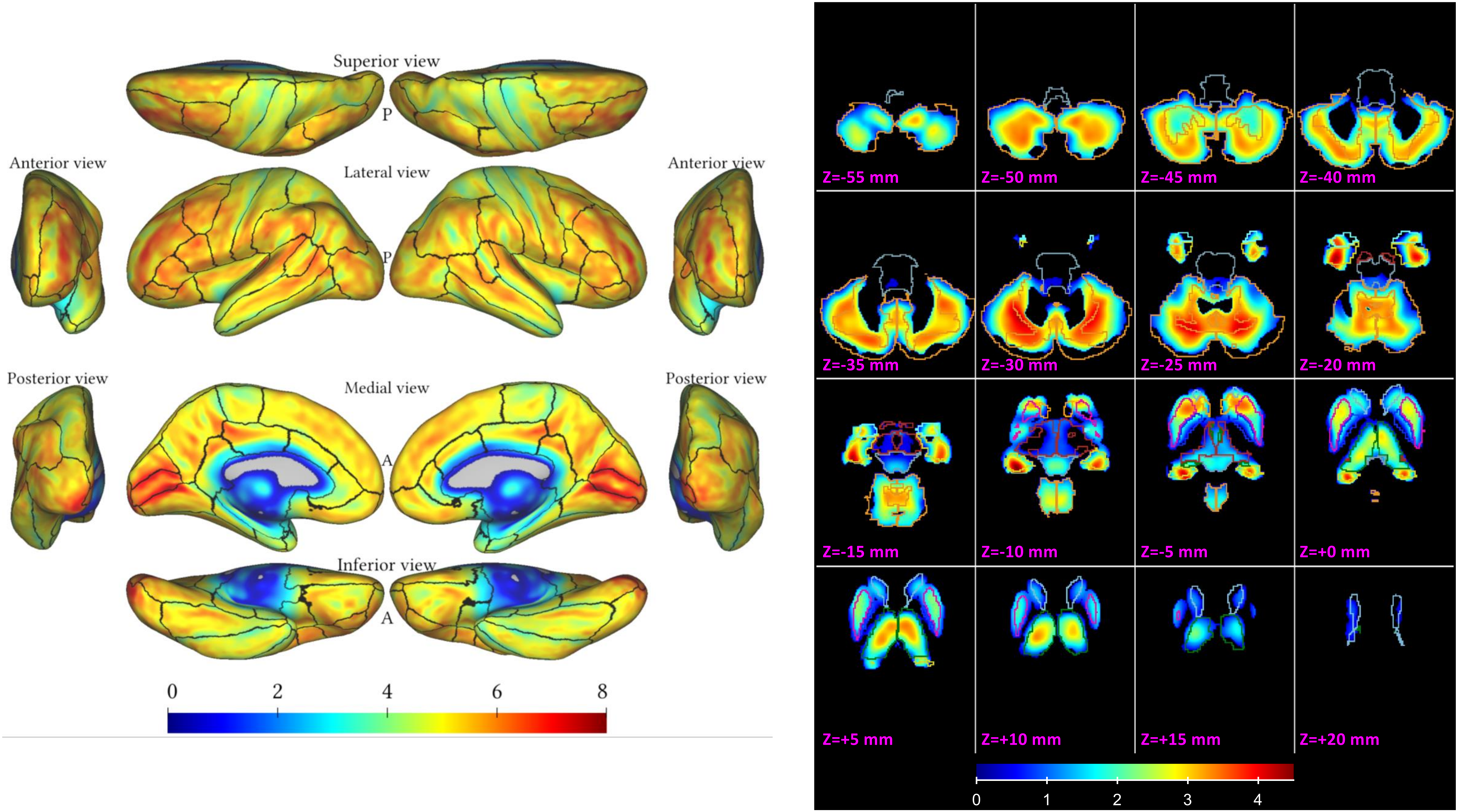

Supplementary Figure S3. Left panel shows [11C]flumazenil binding in the left and right cerebral cortex displayed on the 3d inflated cortical surfaces. Right panel shows [11C]flumazenil binding in subcortical areas displayed on a montage of axial slices. The Z coordinate (axial distance in mm from the plane of the anterior commissure) is indicated for each slice. Boundary lines were transferred from supplemental Fig. S4.

Anatomical structures

Desikan-Killiany atlas cortical parcels

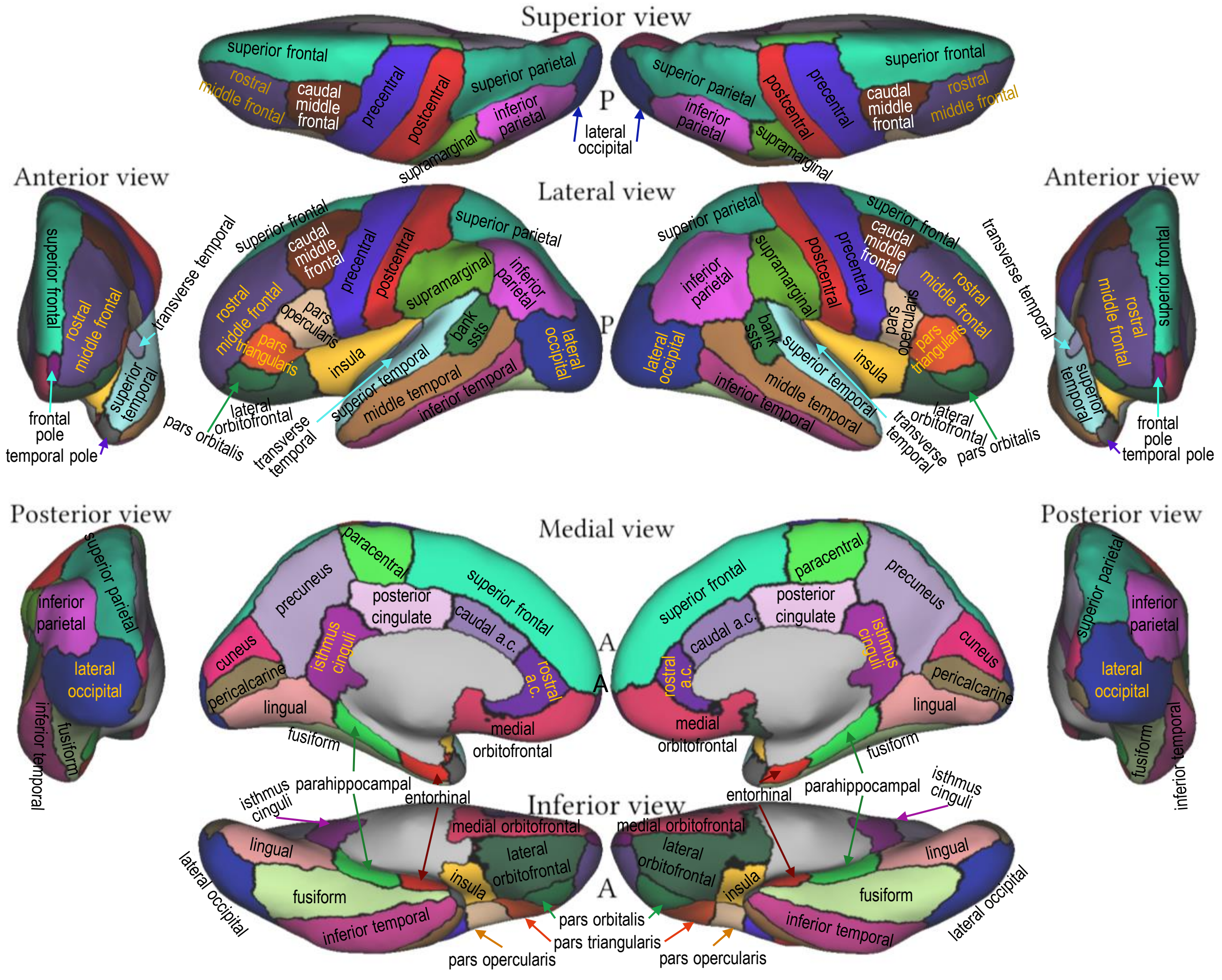

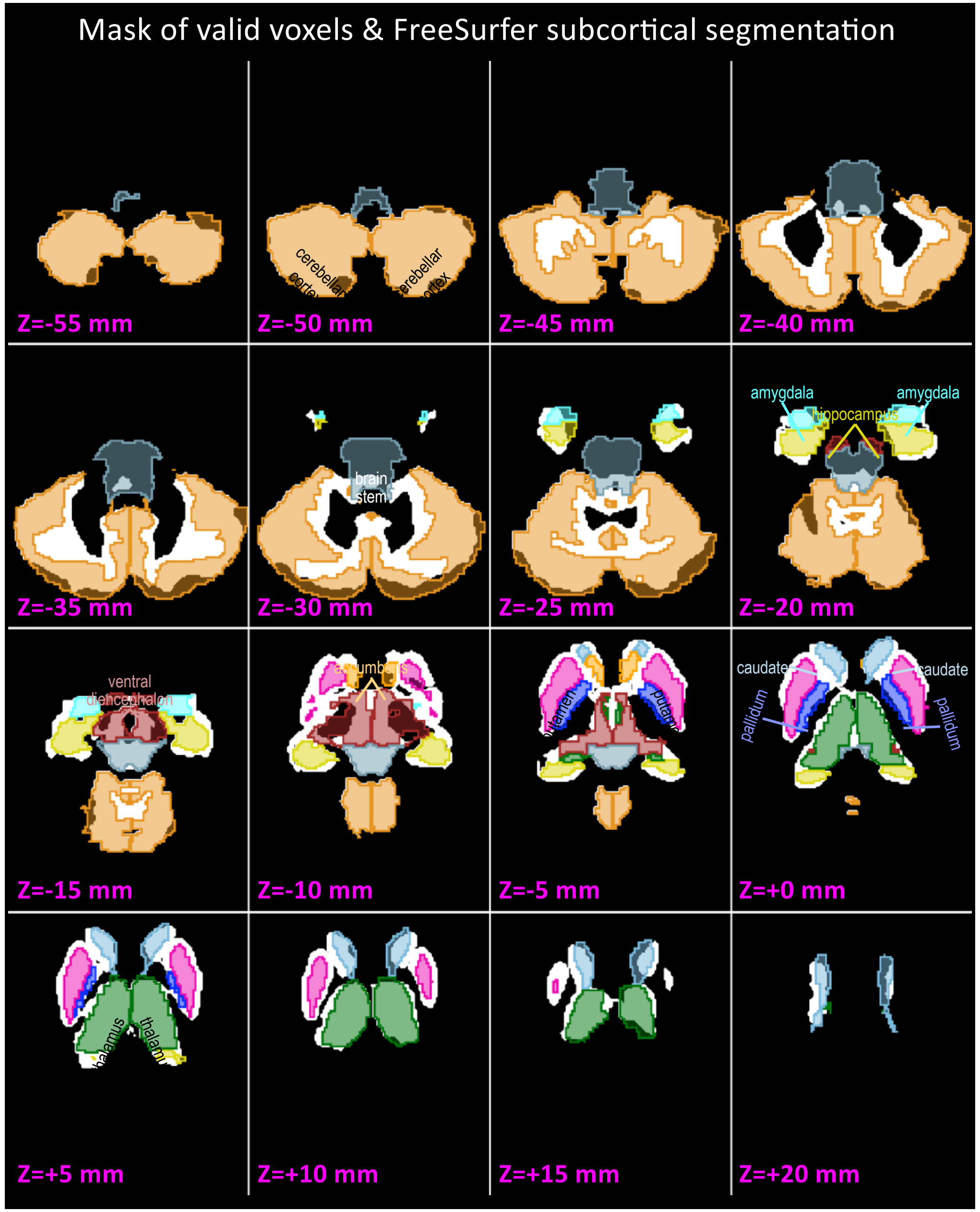

Supplementary Figure S4. Left panel shows anatomical parcellation of the cortical surface according to the Desikan-Killiany atlas in FreeSurfer with names of the cortical parcels. Right panel shows the mask of valid voxels (see step 6. in Fig. 2) in white overlaid with anatomical segmentation of subcortical areas according to FreeSurfer with names and colors of the relevant structures. The names are only shown once for each structure per side in a slice where the structure has a large cross section. The Z coordinate (axial distance in mm from the plane of the anterior commissure) is indicated for each slice.

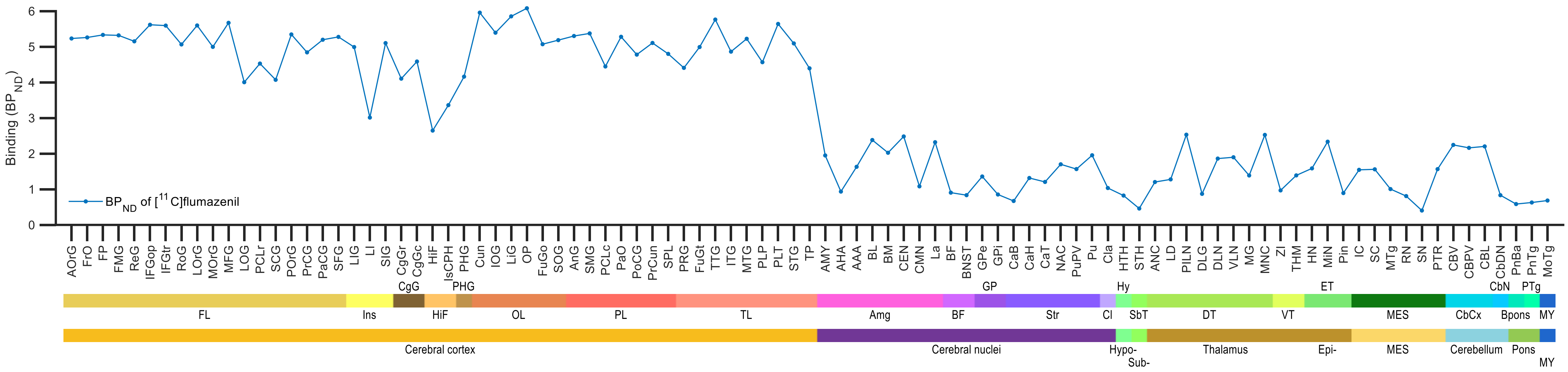

Supplementary Figure S5. Inter-individual average [^{11}C]flumazenil binding (N=12) in substructures defined according to the Allen Human Reference Atlas (AHRA). The substructures are ordered in rostro-caudal direction. Major brain region and structural classification according to the AHRA is indicated at the bottom with abbreviations and colored stripes. FL: frontal lobe, Ins: insula, CgG: cingulate gyrus, HiF: hippocampal formation, PHG: parahippocampal gyrus, OL: occipital lobe, PL: Parietal lobe, TL: temporal lobe, Amg: amygdala, BF: basal forebrain, GP: globus pallidus, Str: striatum, Cl: claustrum, Hy: hypothalamus, SbT: subthalamus, DT: dorsal thalamus, VT: ventral thalamus, ET: epithalamus, MES: mesencephalon, CbCx: cerebellar cortex, CbN: cerebellar nuclei, Bpons: basal part of the pons, PTg: pontine tegmentum, MY: myelencephalon. See substructure names and abbreviations in columns 7 and 8 of Supplementary file 2, respectively.

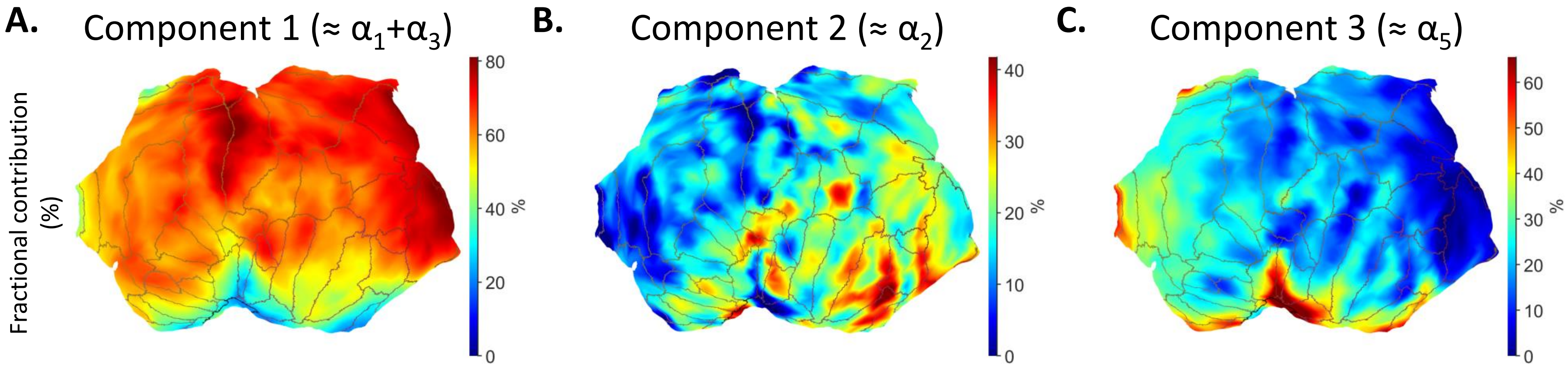

Supplementary Figure S6. (A-C) Component-specific fractional contribution to total [^{11}C]flumazenil binding in the left cerebral cortex displayed on the flattened cortical surface. Anatomical parcellation of the cortical surface according to the AHRA is indicated with boundary lines. The tentative correspondence of the model-specific binding components to $GABA_A$ receptor α subunit expression, based on similarity of their inter-regional pattern to that of gene expression data, is shown in parenthesis for each component.

Fractional contribution (%) of component 1 ($\approx \alpha_1+\alpha_3$)

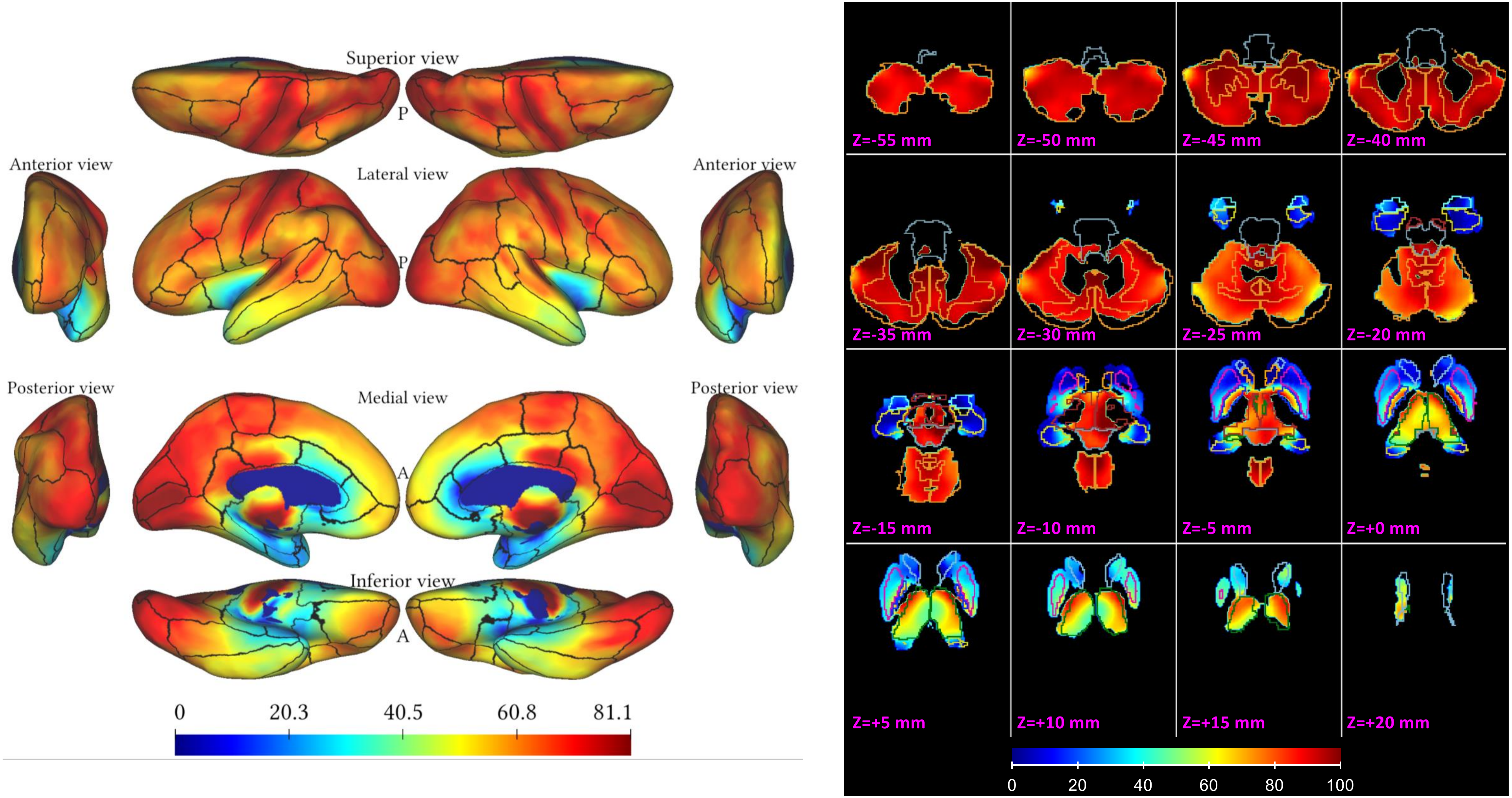

Supplementary Figure S7. Component-1-specific fractional contribution to total [^{11}C]flumazenil binding in the left and right cerebral cortex displayed on the 3d inflated cortical surfaces (left panel) and in subcortical areas displayed on a montage of axial slices (right panel). For the subcortical areas, the Z coordinate (axial distance in mm from the plane of the anterior commissure) is indicated for each slice. Anatomical parcellation of the cortical surface according to the Desikan-Killiany atlas in FreeSurfer, and anatomical segmentation of subcortical areas according to FreeSurfer is indicated with boundary lines (see supplementary Fig. S4). The tentative correspondence of the model-specific binding components to GABA$_A$ receptor α subunit expression, based on similarity of their inter-regional pattern to that of gene expression data, is shown in parenthesis.

Fractional contribution (%) of component 2 (≈ α_2)

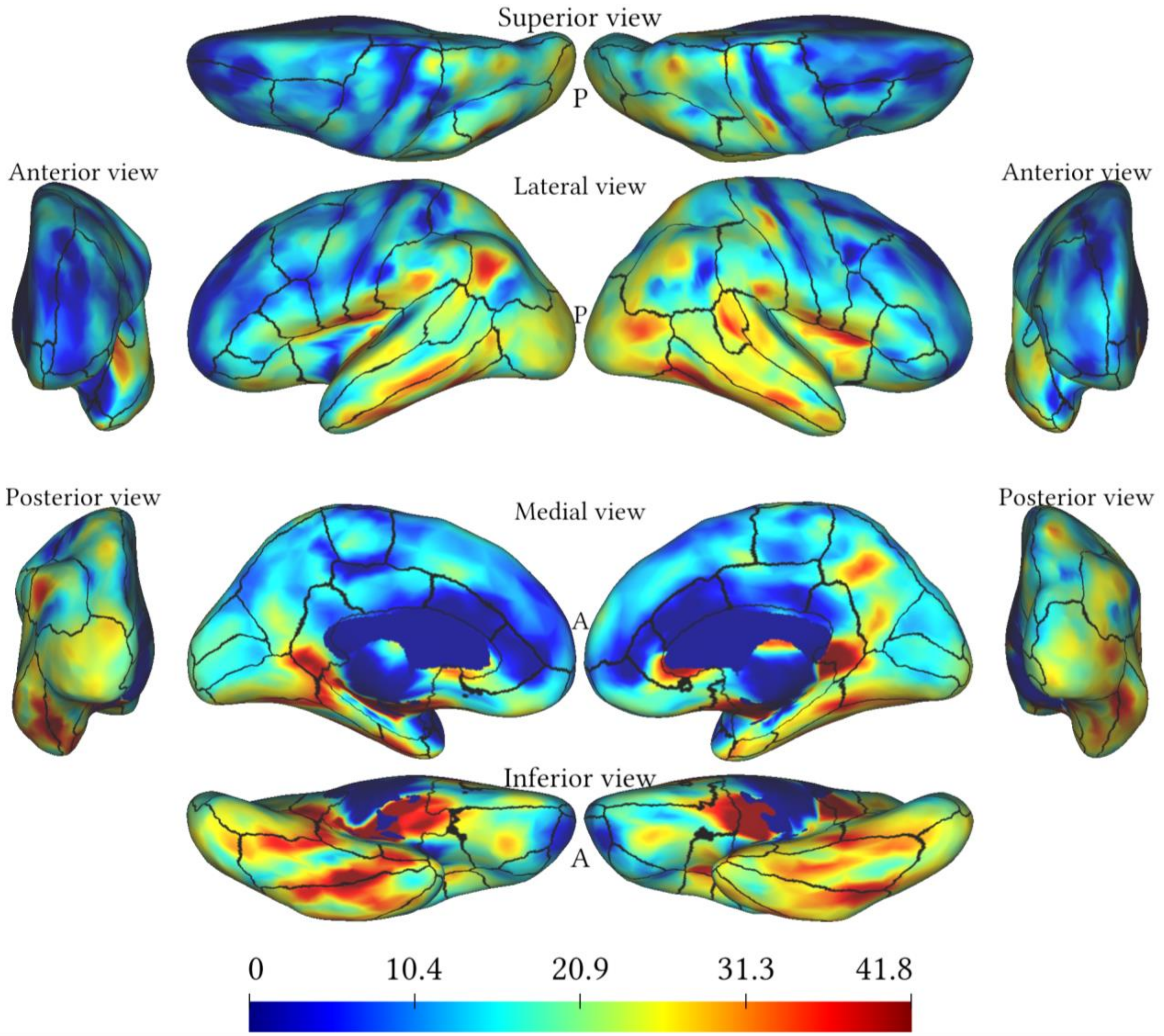

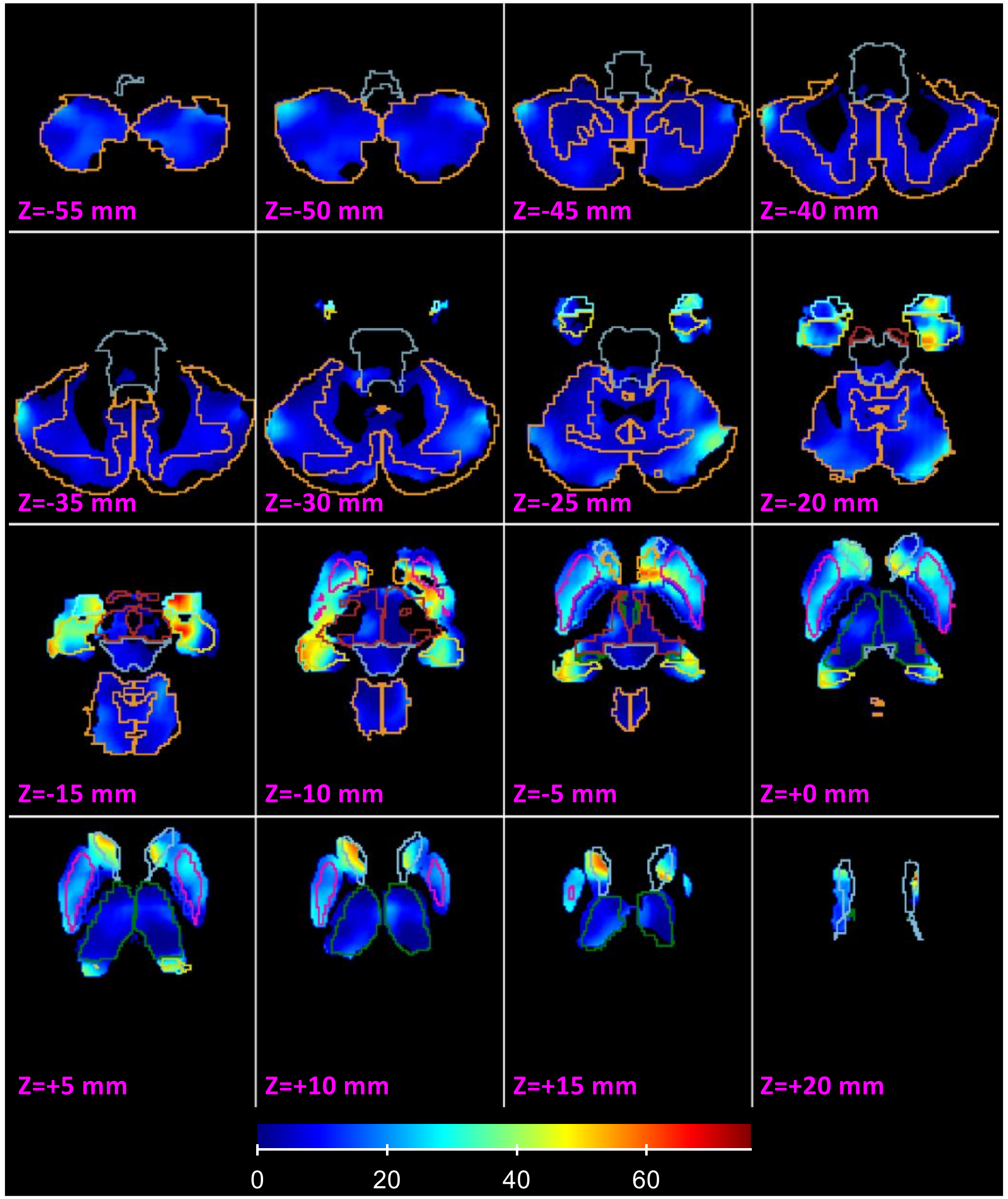

Supplementary Figure S8. Component-2-specific fractional contribution to total [^{11}C]flumazenil binding in the left and right cerebral cortex displayed on the 3d inflated cortical surfaces (left panel) and in subcortical areas displayed on a montage of axial slices (right panel). For the subcortical areas, the Z coordinate (axial distance in mm from the plane of the anterior commissure) is indicated for each slice. Anatomical parcellation of the cortical surface according to the Desikan-Killiany atlas in FreeSurfer, and anatomical segmentation of subcortical areas according to FreeSurfer is indicated with boundary lines (see supplementary Fig. S4). The tentative correspondence of the model-specific binding components to GABA$_A$ receptor α subunit expression, based on similarity of their inter-regional pattern to that of gene expression data, is shown in parenthesis.

Fractional contribution (%) of component 3 (≈ α_5)

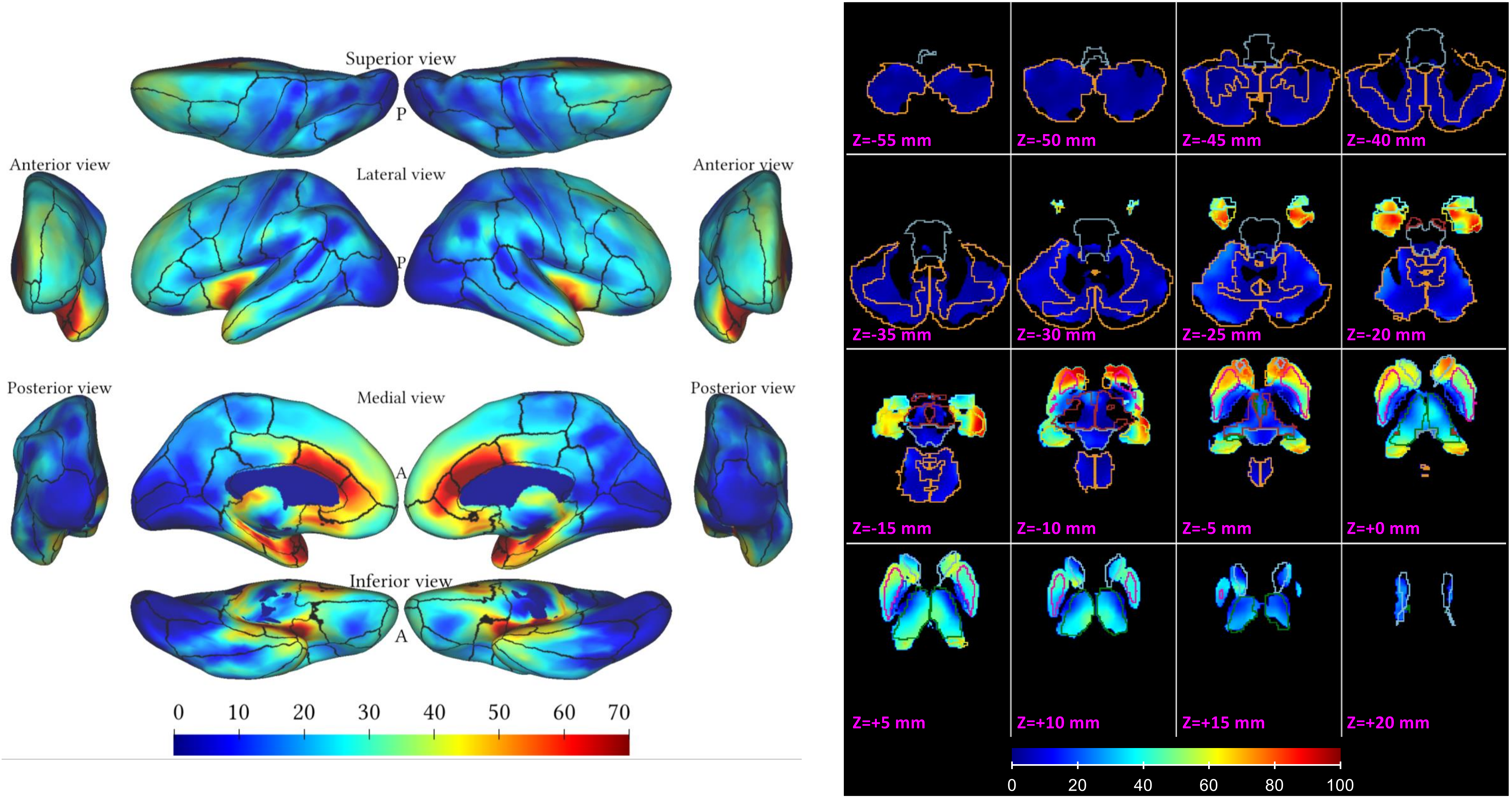

Supplementary Figure S9. Component-3-specific fractional contribution to total [^{11}C]flumazenil binding in the left and right cerebral cortex displayed on the 3d inflated cortical surfaces (left panel) and in subcortical areas displayed on a montage of axial slices (right panel). For the subcortical areas, the Z coordinate (axial distance in mm from the plane of the anterior commissure) is indicated for each slice. Anatomical parcellation of the cortical surface according to the Desikan-Killiany atlas in FreeSurfer, and anatomical segmentation of subcortical areas according to FreeSurfer is indicated with boundary lines (see supplementary Fig. S4). The tentative correspondence of the model-specific binding components to $GABA_A$ receptor α subunit expression, based on similarity of their inter-regional pattern to that of gene expression data, is shown in parenthesis.

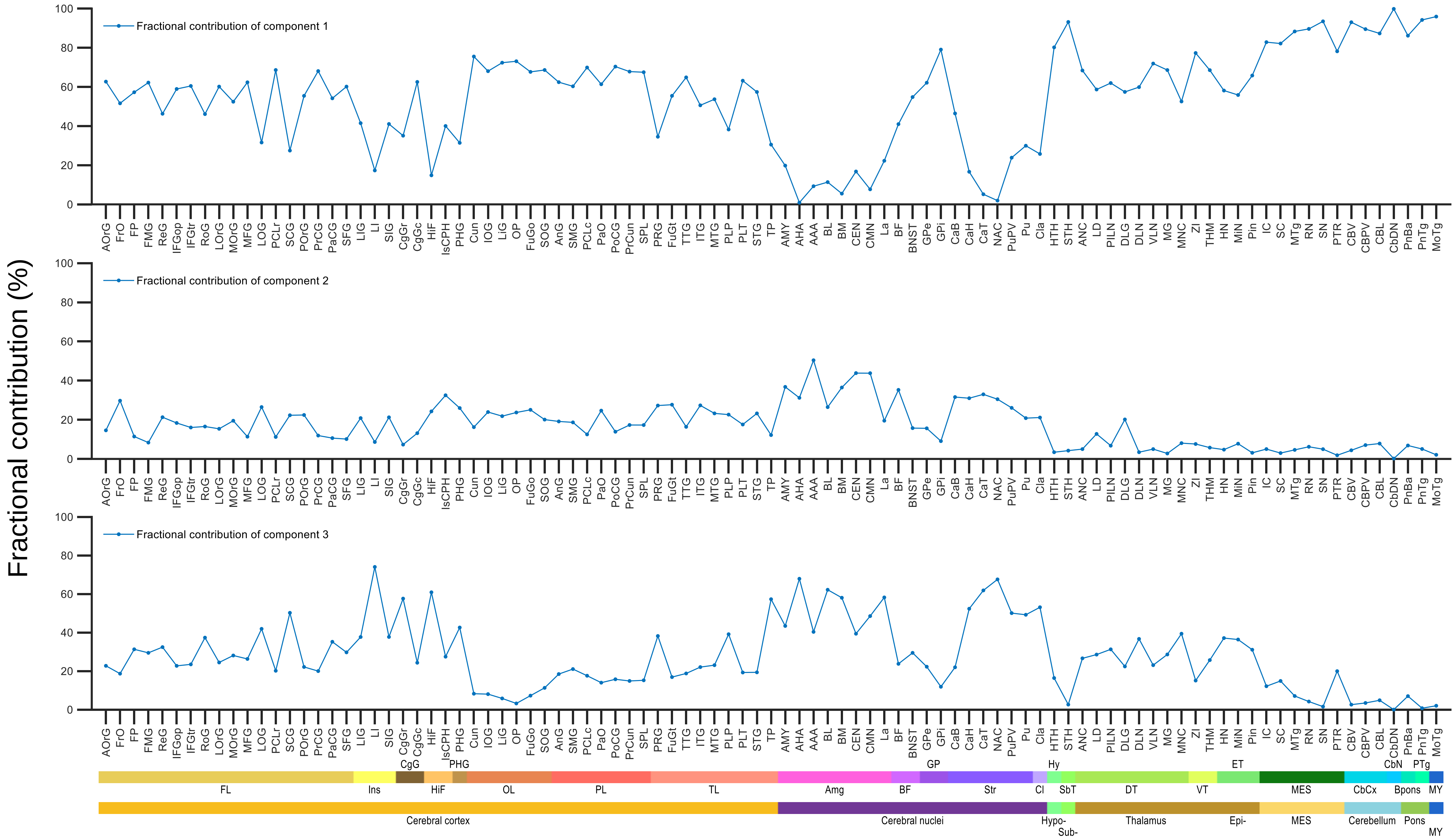

Supplementary Figure S10. Fractional (%) ontribution of the three model derived components (C1-C3) to overall [^{11}C]flumazenil binding in substructures defined according to the Allen Human Reference Atlas (AHRA). The substructures are ordered in rostro-caudal direction. Major brain region and structural classification according to the AHRA is indicated at the bottom with abbreviations and colored stripes. FL: frontal lobe, Ins: insula, CgG: cingulate gyrus, HiF: hippocampal formation, PHG: parahippocampal gyrus, OL: occipital lobe, PL: Parietal lobe, TL: temporal lobe, Amg: amygdala, BF: basal forebrain, GP: globus pallidus, Str: striatum, Cl: claustrum, Hy: hypothalamus, SbT: subthalamus, DT: dorsal thalamus, VT: ventral thalamus, ET: epithalamus, MES: mesencephalon, CbCx: cerebellar cortex, CbN: cerebellar nuclei, Bpons: basal part of the pons, PTg: pontine tegmentum, MY: myelencephalon. See substructure names and abbreviations in columns 7 and 8 of Supplementary file 2, respectively.

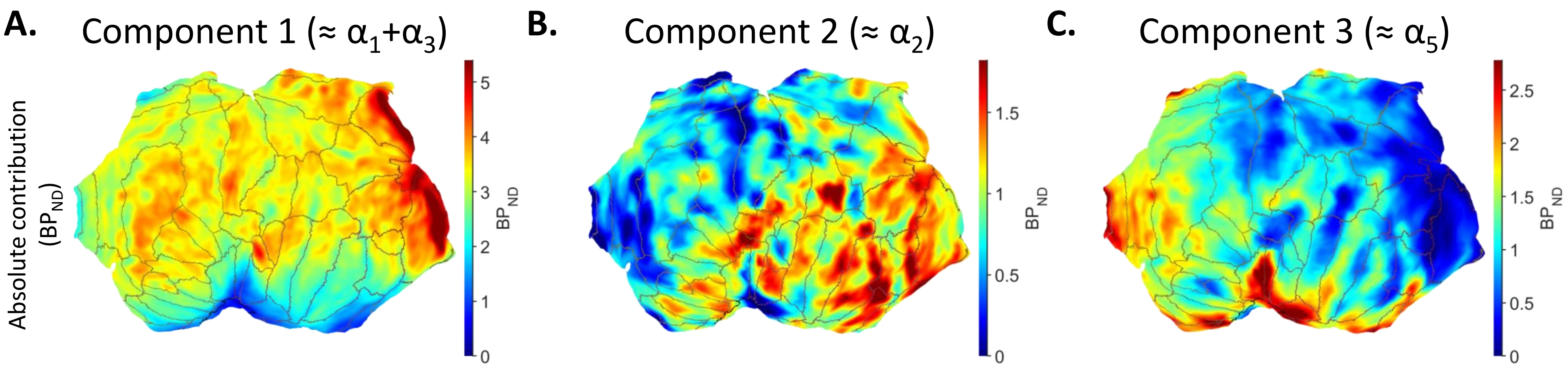

Supplementary Figure S11. (A-C) Component-specific absolute contribution to total [^{11}C]flumazenil binding in the left cerebral cortex displayed on the flattened cortical surface. Anatomical parcellation of the cortical surface according to the AHRA is indicated with boundary lines. The tentative correspondence of the model-specific binding components to $GABA_A$ receptor α subunit expression, based on similarity of their inter-regional pattern to that of gene expression data, is shown in parenthesis for each component.

Absolute contribution (BP_{ND}) of component 1 ($\approx \alpha_1+\alpha_3$)

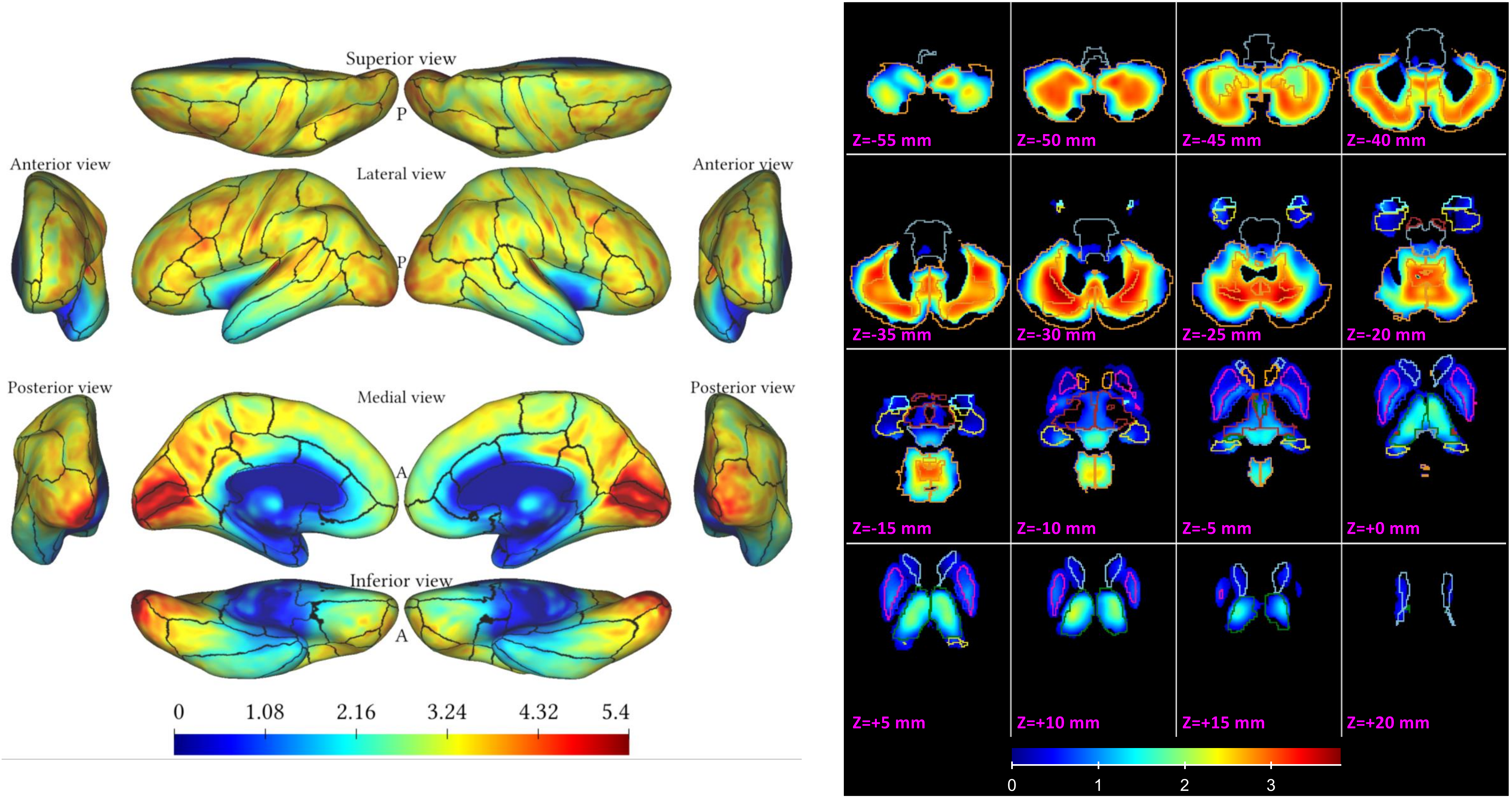

Supplementary Figure S12. Component-1-specific absolute contribution to total [^{11}C]flumazenil binding in the left and right cerebral cortex displayed on the 3d inflated cortical surfaces (left panel) and in subcortical areas displayed on a montage of axial slices (right panel). For the subcortical areas, the Z coordinate (axial distance in mm from the plane of the anterior commissure) is indicated for each slice. Anatomical parcellation of the cortical surface according to the Desikan-Killiany atlas in FreeSurfer, and anatomical segmentation of subcortical areas according to FreeSurfer is indicated with boundary lines (see supplementary Fig. S4). The tentative correspondence of the model-specific binding components to $GABA_A$ receptor α subunit expression, based on similarity of their inter-regional pattern to that of gene expression data, is shown in parenthesis.

Absolute contribution (BP_{ND}) of component 2 (≈ α_2)

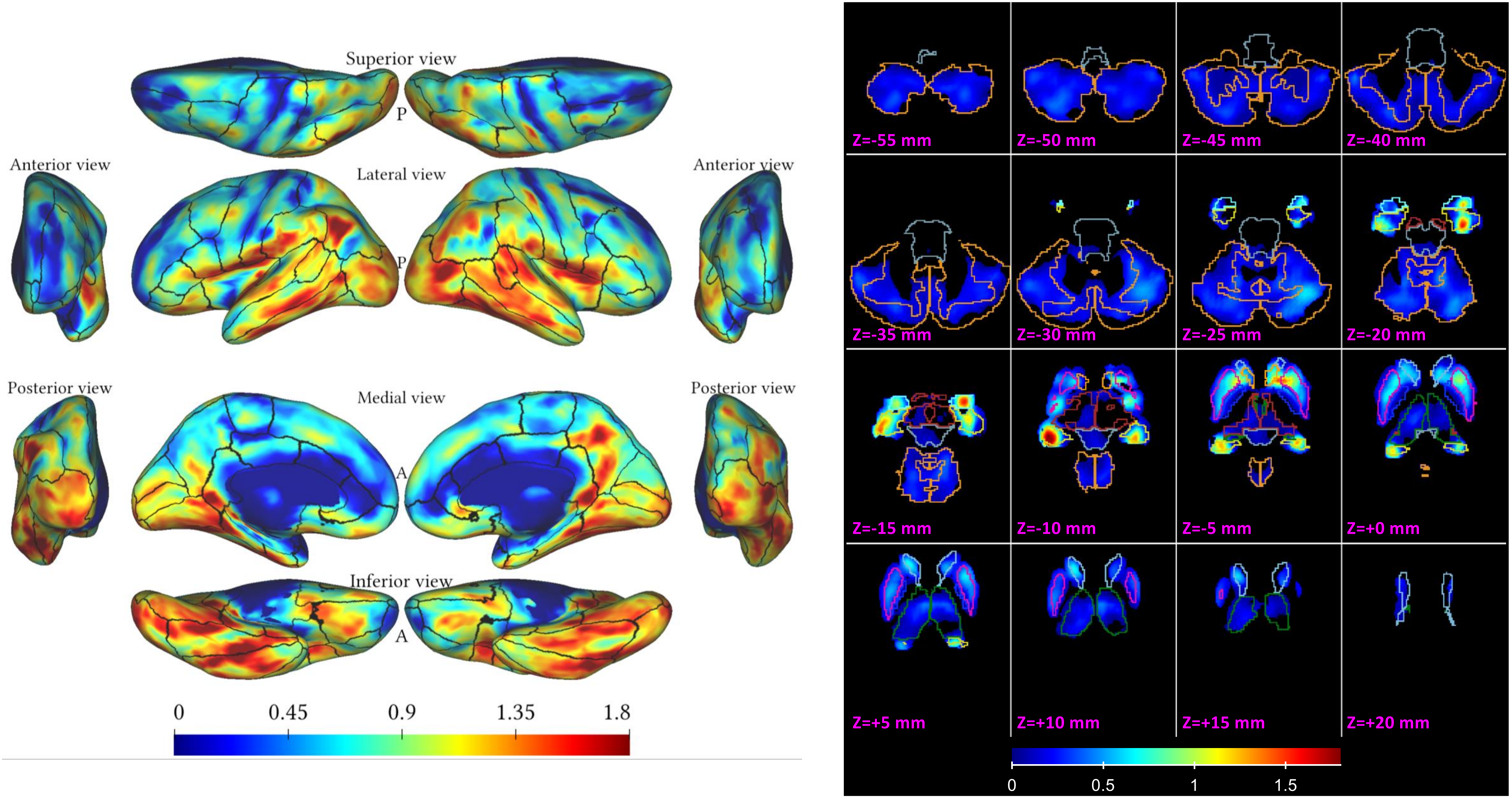

Supplementary Figure S13. Component-2-specific absolute contribution to total [^{11}C]flumazenil binding in the left and right cerebral cortex displayed on the 3d inflated cortical surfaces (left panel) and in subcortical areas displayed on a montage of axial slices (right panel). For the subcortical areas, the Z coordinate (axial distance in mm from the plane of the anterior commissure) is indicated for each slice. Anatomical parcellation of the cortical surface according to the Desikan-Killiany atlas in FreeSurfer, and anatomical segmentation of subcortical areas according to FreeSurfer is indicated with boundary lines (see supplementary Fig. S4). The tentative correspondence of the model-specific binding components to $GABA_A$ receptor α subunit expression, based on similarity of their inter-regional pattern to that of gene expression data, is shown in parenthesis.

Absolute contribution (BP_{ND}) of component 3 (≈ α_5)

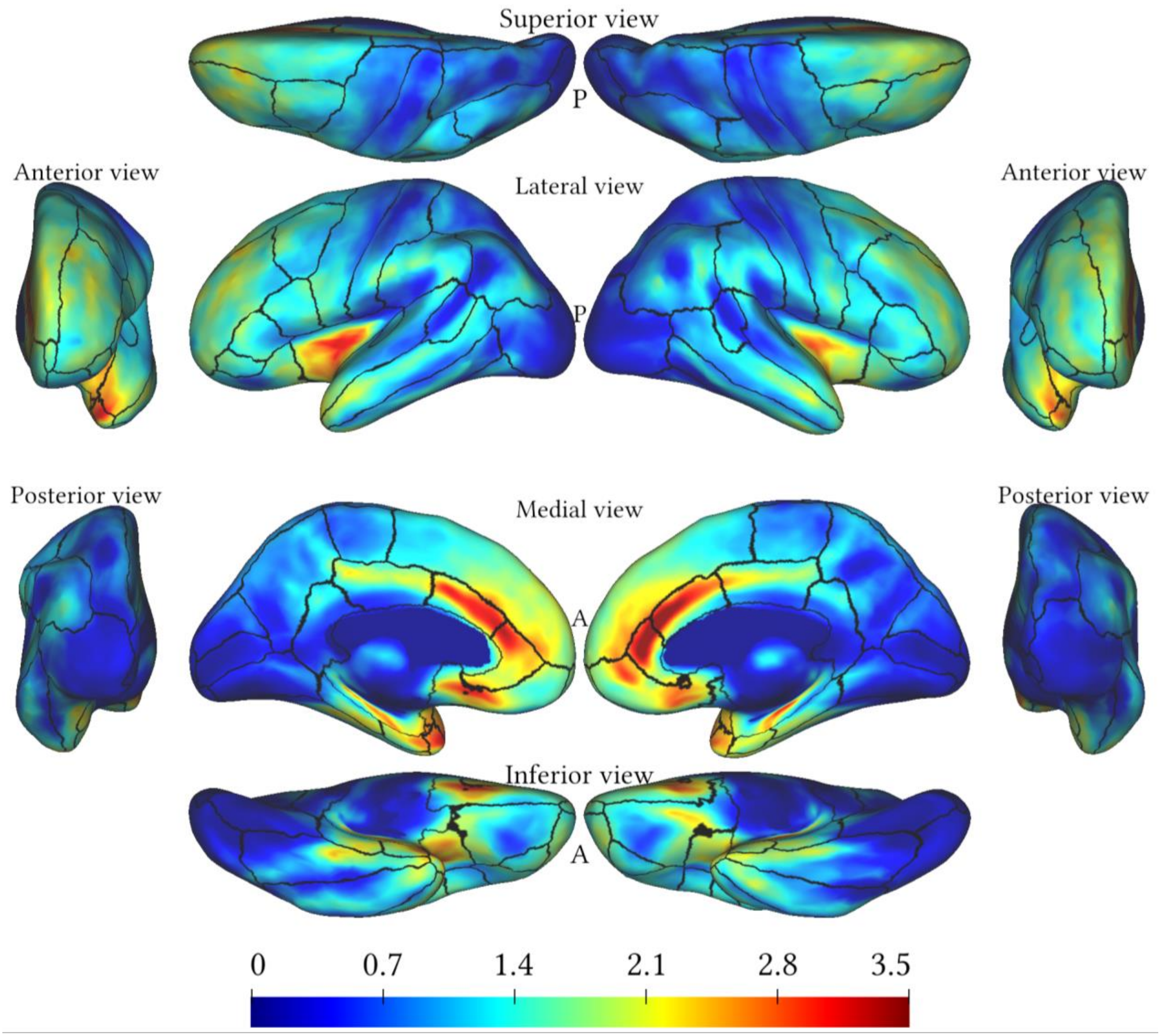

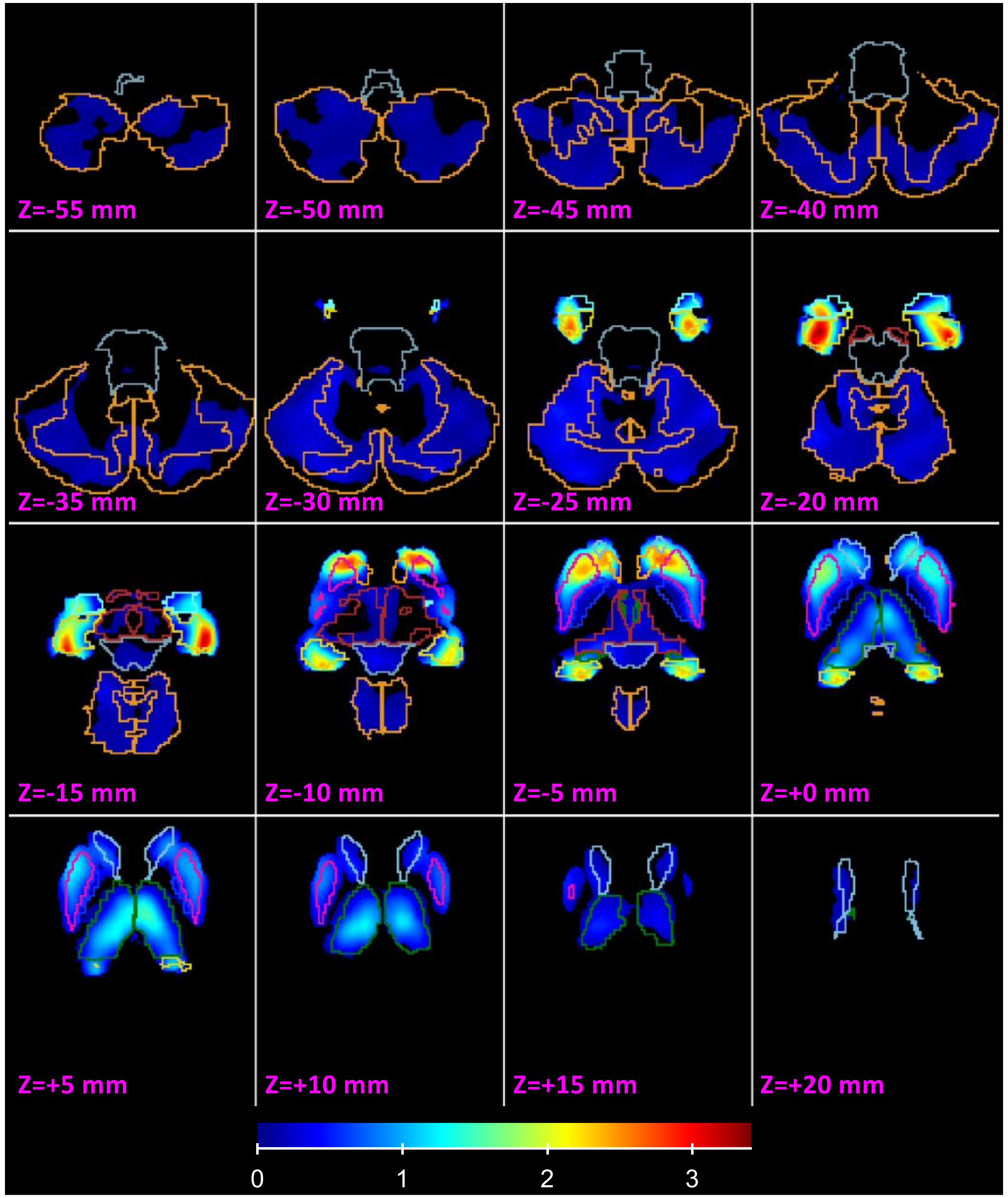

Supplementary Figure S14. Component-3-specific absolute contribution to total [^{11}C]flumazenil binding in the left and right cerebral cortex displayed on the 3d inflated cortical surfaces (left panel) and in subcortical areas displayed on a montage of axial slices (right panel). For the subcortical areas, the Z coordinate (axial distance in mm from the plane of the anterior commissure) is indicated for each slice. Anatomical parcellation of the cortical surface according to the Desikan-Killiany atlas in FreeSurfer, and anatomical segmentation of subcortical areas according to FreeSurfer is indicated with boundary lines (see supplementary Fig. S4). The tentative correspondence of the model-specific binding components to $GABA_A$ receptor α subunit expression, based on similarity of their inter-regional pattern to that of gene expression data, is shown in parenthesis.

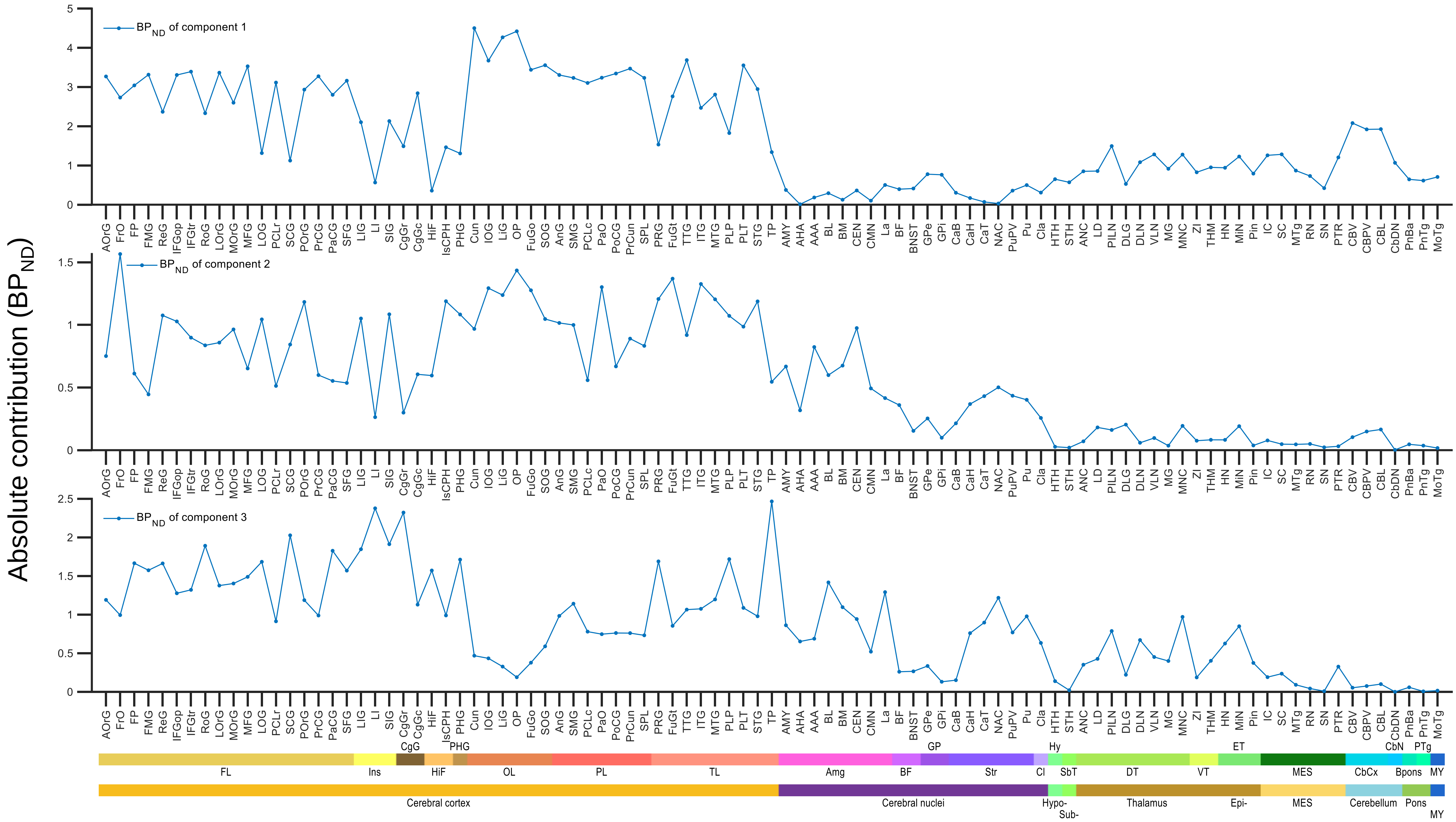

Supplementary Figure S15. Inter-individual average [^{11}C]flumazenil binding (N=12, top chart), and absolute (BP_{ND}) contribution of the three model derived components (C1-C3) to overall [^{11}C]flumazenil binding (bottom three charts, respectively) in substructures defined according to the Allen Human Reference Atlas (AHRA). The substructures are ordered in rostro-caudal direction. Major brain region and structural classification according to the AHRA is indicated at the bottom with abbreviations and colored stripes. FL: frontal lobe, Ins: insula, CgG: cingulate gyrus, HiF: hippocampal formation, PHG: parahippocampal gyrus, OL: occipital lobe, PL: Parietal lobe, TL: temporal lobe, Amg: amygdala, GP: globus pallidus, Str: striatum, Cl: claustrum, Hy: hypothalamus, SbT: subthalamus, DT: dorsal thalamus, VT: ventral thalamus, MES: mesencephalon, CbCx: cerebellar cortex, CbN: cerebellar nuclei, Bpons: basal part of the pons, PTg: pontine tegmentum, MY: myelencephalon. See substructure names and abbreviations in columns 7 and 8 of Supplementary file 2, respectively.

Supplementary Figure S16.

Estimated regional fractional (%) contribution of the 3 model-derived components (C1-C3) to total [^{11}C]flumazenil binding in comparison to published gene expression data (Hawrylycz et al. 2012) in substructures defined according to the Allen Human Reference Atlas (AHRA). The substructures are ordered in rostro-caudal direction. Gene expression is the proportion of specific $GABA_A$ receptor α subunit mRNA expression in percent of the summed expression of the α_1, α_2, α_3 and α_5 subunits. For each component, the charts show selected comparisons guided by observed correlations between respective model-predicted component fractional contributions and specific α subunit expressions. In detail, in case of component 1 the comparison to the fractional expression of GABRA1 (R=0.80 [cross-validation set R=0.80±0.011, 0.78 – 0.83], R^2=0.64, corr. p-value=1×10^{-14}), GABRA3 (R=0.303 [cross-validation set R=0.304±0.011, 0.28 – 0.32], R^2=0.09, p-value=0.017), and to the summed fractional expression of GABRA1 and GABRA3 (R=0.87 [cross-validation set R=0.87±0.009, 0.85 – 0.89], R^2=0.75, corr. p-value=5×10^{-19}) are shown. In case of component 2, the comparison to the fractional expression of GABRA2 (R=0.71 [cross-validation set R=0.66±0.082, 0.43 – 0.77], R^2=0.51, corr. p-value=2×10^{-10}) is shown. And in case of component 3, the comparison to the fractional expression of GABRA5 (R=0.81 [cross-validation set R=0.80±0.011, 0.77 – 0.81], R^2=0.65, corr. p-value=1×10^{-14}) is shown. Major brain region and structural classification according to the AHRA is indicated at the bottom with abbreviations and colored stripes. FL: frontal lobe, Ins: insula, CgG: cingulate gyrus, HiF: hippocampal formation, PHG: parahippocampal gyrus, OL: occipital lobe, PL: Parietal lobe, TL: temporal lobe, Amg: amygdala, BF: basal forebrain, GP: globus pallidus, Str: striatum, Cl: claustrum, Hy: hypothalamus, SbT: subthalamus, DT: dorsal thalamus, VT: ventral thalamus, MES: mesencephalon, CbCx: cerebellar cortex, CbN: cerebellar nuclei, Bpons: basal part of the pons, PTg: pontine tegmentum, MY: myelencephalon. See substructure names and abbreviations in columns 7 and 8 of Supplementary file 2, respectively.

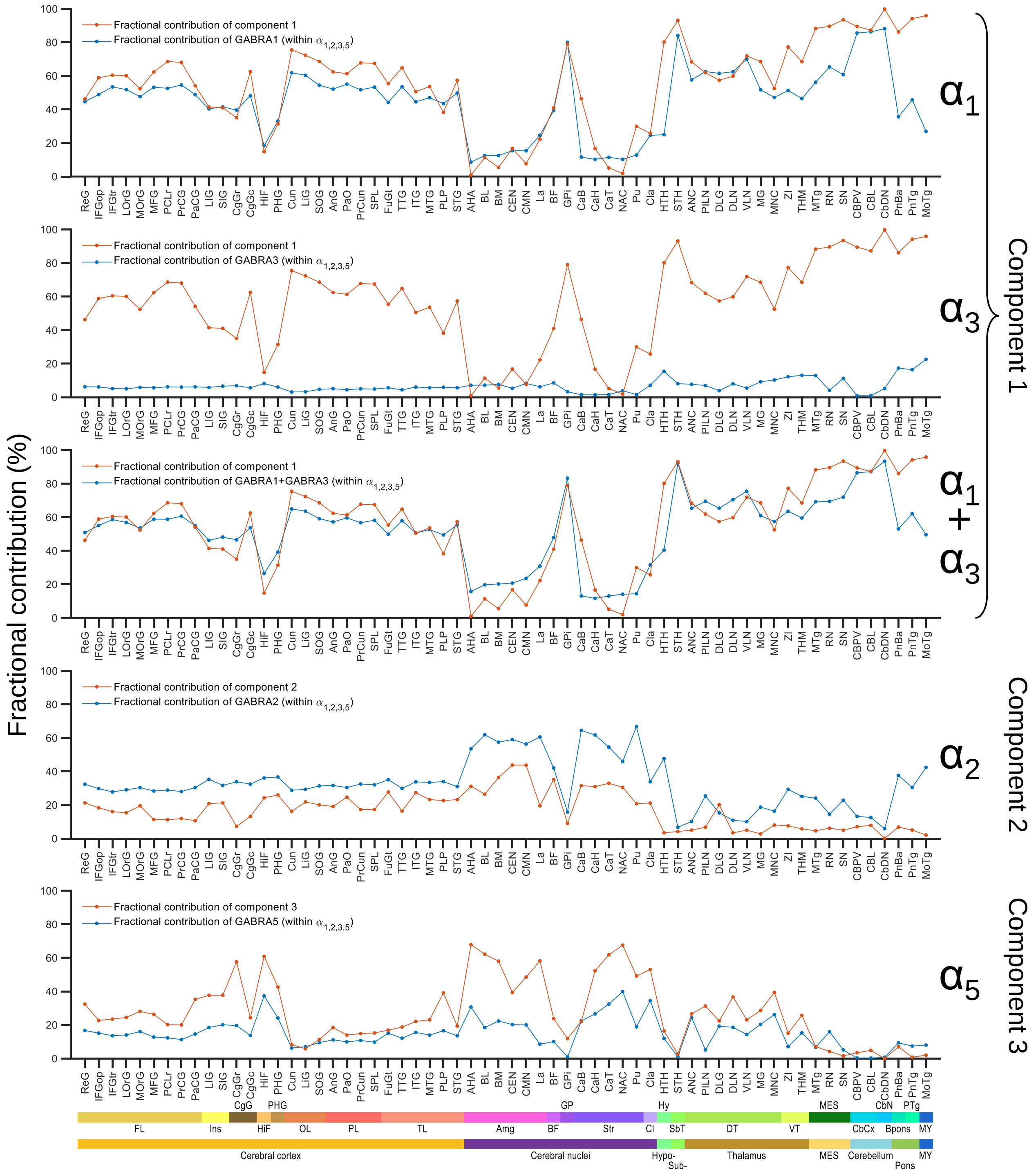

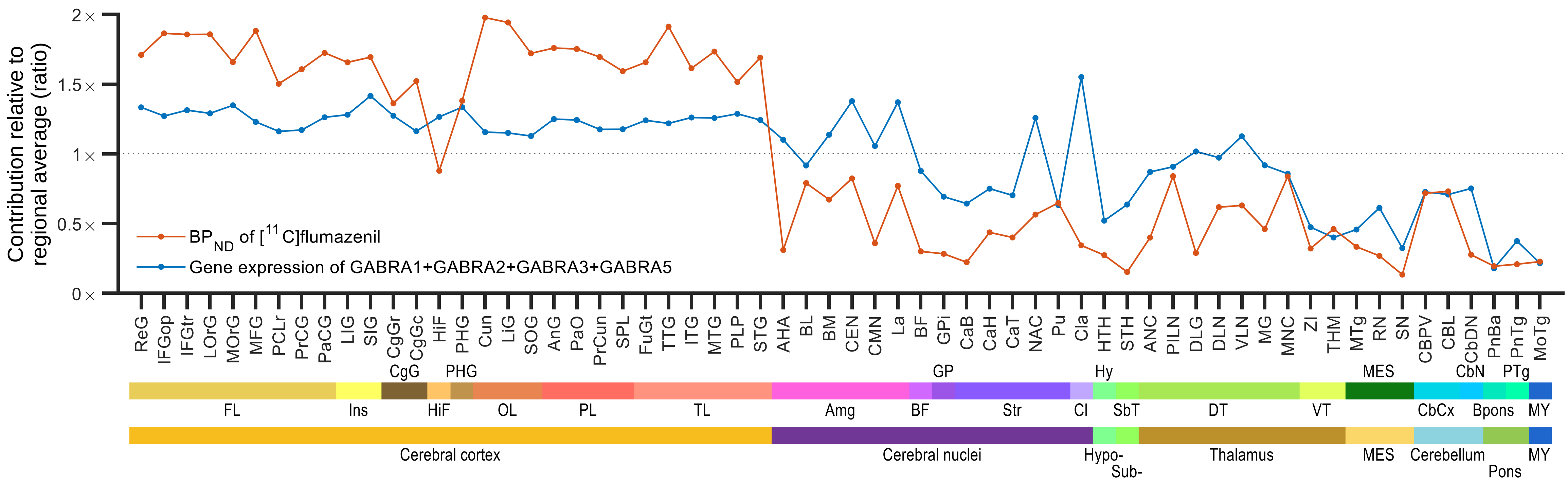

Supplementary Figure S17.

Comparison of AHRA substructure-wise $GABA_A$ receptor α subunit gene expression (summed expression of GABRA1+GABRA2+GABRA3+GABRA5 mRNA) and [^{11}C]flumazenil binding. There was a high-degree, positive correlation: R=0.72, R^2=0.52, p-val=2.5e-10. Regional values plottted in substructures defined according to the Allen Human Reference Atlas (AHRA). The substructures are ordered in rostro-caudal direction. Major brain region and structural classification according to the AHRA is indicated at the bottom with abbreviations and colored stripes. FL: frontal lobe, Ins: insula, CgG: cingulate gyrus, HiF: hippocampal formation, PHG: parahippocampal gyrus, OL: occipital lobe, PL: Parietal lobe, TL: temporal lobe, Amg: amygdala, BF: basal forebrain, GP: globus pallidus, Str: striatum, Cl: claustrum, Hy: hypothalamus, SbT: subthalamus, DT: dorsal thalamus, VT: ventral thalamus, ET: epithalamus, MES: mesencephalon, CbCx: cerebellar cortex, CbN: cerebellar nuclei, Bpons: basal part of the pons, PTg: pontine tegmentum, MY: myelencephalon. See substructure names and abbreviations in columns 7 and 8 of Supplementary file 2, respectively.

Comparator	R-value	corr. p-value
absGABRA1235	0.72	2.51048e-10

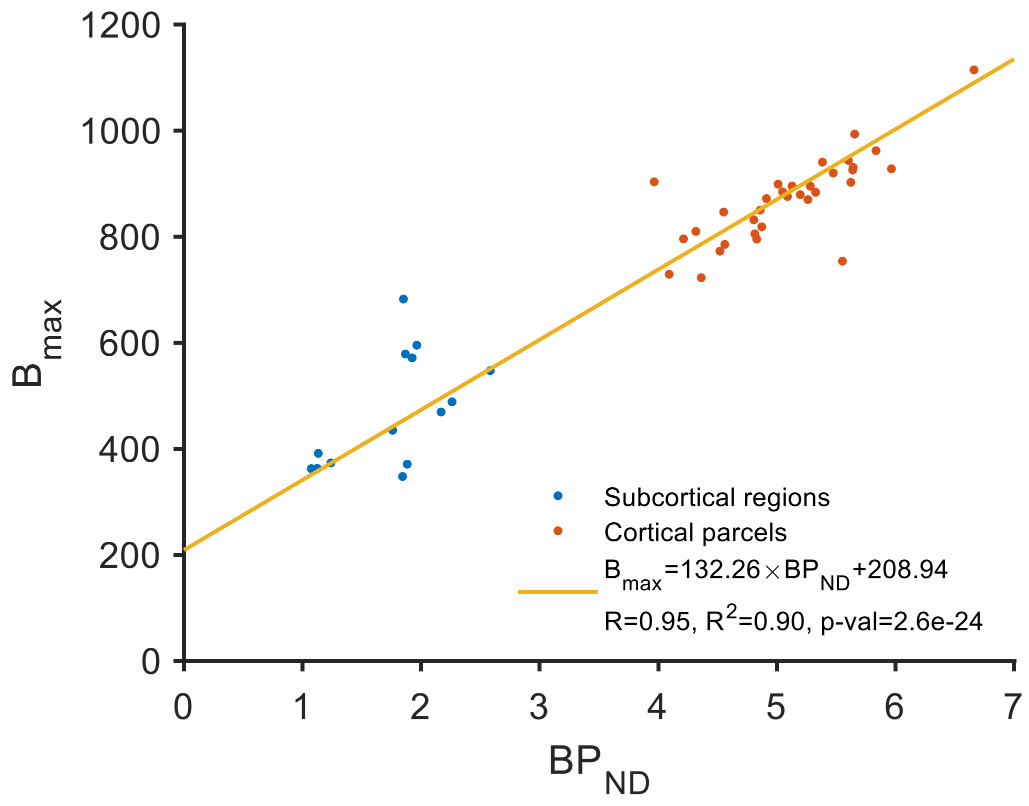

Supplementary Figure S18.

Regression plot of regional [^{11}C]flumazenil B_{max} values reported previously (Nørgaard et al., 2021, N=16) and inter-subject average [^{11}C]flumazenil BP_{ND} in our data (N=12). Regions used: Desikan-Killiany cortical parcellation and subcortical segmentation (cerebral nuclei + thalamus) according to FreeSurfer.
